# A universal kinetic framework for quantitative isothermal amplification governed by polymerase speed, amplicon size, and binding efficiency

**DOI:** 10.1093/nar/gkaf1378

**Published:** 2026-01-06

**Authors:** Langjun Tang, Zhenyu Guo, Jinyong Wu, Yonghong Li, Kun Yang

**Affiliations:** Department of Pharmaceutical & Biological Engineering, School of Chemical Engineering, Sichuan University, Chengdu 610065, China; Department of Pharmaceutical & Biological Engineering, School of Chemical Engineering, Sichuan University, Chengdu 610065, China; Department of Pharmaceutical & Biological Engineering, School of Chemical Engineering, Sichuan University, Chengdu 610065, China; Department of Pharmaceutical & Biological Engineering, School of Chemical Engineering, Sichuan University, Chengdu 610065, China; Department of Pharmaceutical & Biological Engineering, School of Chemical Engineering, Sichuan University, Chengdu 610065, China

## Abstract

The lack of a predictive, first-principles model has confined the development of isothermal exponential amplification (IEA) to empirical optimization for decades, hindering its quantitative potential. Here, we resolve this by establishing a universal kinetic framework that defines amplification efficiency through three fundamental physical parameters: the polymerase extension rate (*S*_*e*_), the amplicon size (*S*_*a*_), and the primer-template binding efficiency (*ξ*). The model reveals the apparent doubling time as $T = \frac{{{{S}_a}}}{{\xi \cdot {{S}_e}}}$, providing a unified physical explanation for IEA efficiency across diverse mechanisms, including loop-mediated isothermal amplification (LAMP), strand displacement amplification (SDA), recombinase polymerase amplification (RPA), and helicase-dependent amplification (HDA). Crucially, we mathematically demonstrate that the complex kinetics of LAMP, a case study with extreme product heterogeneity, are structurally isomorphic to simple exponential growth. A Taylor expansion proves that LAMP product heterogeneity emerges as a Poisson process, a prediction we confirm experimentally. This framework accurately predicts quantification outcomes under varying conditions (enzyme, temperature, and inhibitors) and enables robust viral quantification in wastewater. By bridging fundamental enzymology with point-of-care applications, our work provides a general blueprint for optimizing IEA by engineering polymerase speed (*S*_*e*_), minimizing amplicon size (*S*_*a*_), and fine-tuning primer-binding efficiency (*ξ*).

## Graphical Abstract

**Graphical Abstract:**
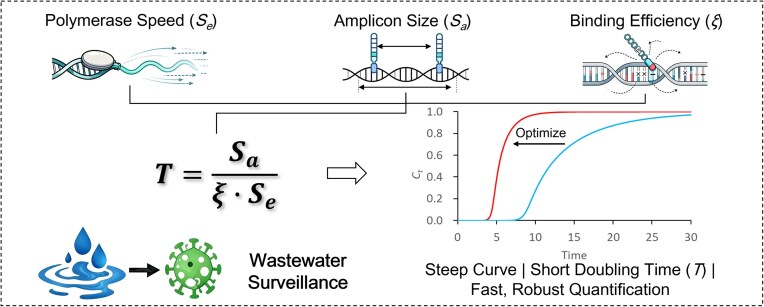

## Significance statement

Isothermal nucleic acid amplification techniques promise rapid, equipment-free molecular diagnostics but suffer from unreliable quantification. We address the long-standing challenge by introducing a kinetic framework of isothermal exponential amplification (IEA) based on physically meaningful parameters: polymerase speed, amplicon length, and primer-binding efficiency. The model quantifies how these factors determine amplification robustness, explains failures in current methods [e.g. loop-mediated isothermal amplification (LAMP)], and provides actionable optimization strategies. We validate the framework experimentally and demonstrate its utility in wastewater-based epidemiology. This work bridges a critical gap between IEA technology and quantitative applications, promoting next-generation point-of-care (POC) diagnostics.

## Introduction

Since its development, the polymerase chain reaction (PCR) [1] has become ubiquitous in nucleic acid amplification, permeating diverse fields—including recombinant DNA technology, molecular diagnostics, and forensics—that require amplification and detection of nucleic acids at ultra-low initial concentrations. Nucleic acid amplification techniques are broadly categorized as either thermocycling amplification or isothermal amplification, based on whether the reaction involves temperature cycling [2]. PCR, the prototypical thermocycling method, has evolved into quantitative PCR (qPCR) through extensive technical refinement and theoretical study [3], establishing it as the molecular diagnostics gold standard. However, qPCR’s reliance on programmable thermocyclers significantly limits its application in resource-limited settings and POC testing [4].

To overcome this limitation, various isothermal amplification techniques have been developed since the early 1990s [5]. These techniques eliminate the need for expensive thermocyclers and are amenable to integration into microfluidic chips for POC diagnostic devices [2]. Like qPCR, most isothermal methods generate amplification products that serve as templates for further replication, resulting in exponential amplification. Consequently, many IEA techniques, including strand displacement amplification (SDA) [6], nucleic acid sequence-based amplification (NASBA) [7], helicase-dependent amplification (HDA) [8], recombinase polymerase amplification (RPA) [9], and loop-mediated isothermal amplification (LAMP) [10, 11], are considered suitable not only for qualitative detection but also for target nucleic acid quantification. Researchers have hypothesized that IEA should be intrinsically capable of quantification, leading to sustained efforts to develop mathematical models describing its mechanism [6, 12]. Complex stoichiometric and empirical models have been proposed to elucidate LAMP kinetics [13–15]. However, a mathematical model with clearly defined physical significance describing IEA kinetics and its quantification mechanism remains elusive. This gap likely explains why IEA, despite its significant advantages over qPCR (particularly in POC applications), has not supplanted qPCR as the molecular diagnostics gold standard. Achieving comparable diagnostic status to qPCR is a critical goal for IEA.

To address this, we introduce a universal mechanistic framework for IEA that defines the doubling time (*T*) using physically meaningful parameters: polymerase extension rate (*S*_*e*_), amplicon size (*S*_*a*_), and primer-template binding efficiency (*ξ*). This framework resolves the previously unresolved kinetics limiting IEA-based quantification and provides actionable optimization strategies. While we use LAMP as a rigorous experimental validation system due to its complex mechanism, the model is formulated to be applicable across diverse IEA techniques by capturing their shared physical essence.

## Theory

### Universal kinetic model of IEA

We propose a universal kinetic framework that governs the efficiency of any IEA process through three fundamental physical parameters. During the initial stage of IEA, substrates such as primers and dNTP are in excess, and enzyme molecules operate under unsaturated conditions with constant activity. As illustrated in Supplementary Fig. S1A, the ideal doubling time (${{T}^\theta } = \frac{{{{S}_a}}}{{{{S}_e}}}$) corresponds to the time required for a polymerase to fully extend a single amplicon of size *S*_*a*_​ at a rate *S*_*e*​_ (in bp/min). Here, *S*_*e*_ represents the effective nucleic acid polymerase activity under the given reaction conditions. A simplified schematic in Supplementary Fig. S1B omits explicit double-strand unwinding mechanics for clarity.

The kinetics of IEA can be described by the recurrence relation for amplicon copy number *N*_*t*_ (copies/μl):


(1) \begin{eqnarray*}
{{N}_{t + \Delta t}} = {{\left( {1 + \eta } \right)}^{\frac{{\Delta t}}{{{{T}^\theta }}}}}{{N}_t}
\end{eqnarray*}

Under nonideal conditions, the template’s primer-binding rate (*η*) modulates the apparent doubling time *T*. Rewriting Eq. 1 as:


(2) \begin{eqnarray*}
{{N}_{t + \Delta t}} = {{2}^{\frac{{\Delta t}}{T}}}{{N}_t}
\end{eqnarray*}

yields:


(3) \begin{eqnarray*}
{{2}^{\frac{{\Delta t}}{T}}} = {{\left( {1 + \eta } \right)}^{\frac{{\Delta t}}{{{{T}^\theta }}}}}
\end{eqnarray*}

Solving for *T* gives,


(4) \begin{eqnarray*}
T = \frac{{{{T}^\theta }}}{{{{{\log }}_2}\left( {1 + \eta } \right)}}
\end{eqnarray*}

Defining $\xi = {{\log }_2}( {1 + \eta } )$, a dimensionless parameter quantifying primer-template binding efficiency, i.e. specificity and annealing properties, the apparent doubling time simplifies to:


(5) \begin{eqnarray*}
T = \frac{{{{T}^\theta }}}{\xi } = \frac{{{{S}_a}}}{{\xi \cdot {{S}_e}}}
\end{eqnarray*}

The parameter $T = \frac{{{{S}_a}}}{{\xi \cdot {{S}_e}}}$ serves as the pivotal determinant of exponential amplification efficiency, integrating three fundamental physical factors: polymerase activity *S*_*e*_, apparent amplicon size *S*_*a*_, and primer-template binding efficiency *ξ*. This formulation establishes a unified theoretical framework for IEA, applicable to diverse techniques, including LAMP, SDA, RPA, and HDA, despite differences in their strand-displacement mechanisms. Derivations of *T*^θ^ for each mechanism are provided in Supplementary Document S1. Expressions of *T*^θ^ for various IEA mechanisms.

### Physical interpretation of η and ξ

IEA comprises two stages: annealing equilibrium and extension (Supplementary Fig. S1C). During annealing, competitive equilibria arise: the target template self-annealing with equilibrium constant *K*_1_, and the primer-template annealing with equilibrium constant *K*_2_. The strands extension catalyzed by the nucleic acid polymerase promotes the annealing equilibrium to move to the right and enter the next amplification cycle.

Define:

*c*_*p*_ = primer concentration (μM);

*c*_*t*_ = total target concentration at time *t* (μM);

*c*_*t*,ss_ = concentration of single-stranded target (μM);

*c*_*t*,ds_ = concentration of double-stranded target (μM);

*c*_*b*_ = concentration of primer-template complex (μM);

${{K}_1} = {{c}_{t,ds}}/c_{t,ss}^2$ , target template self-annealing constant (μM^−1^);

${{K}_2} = {{c}_b}/{{c}_p} \cdot {{c}_{t,ss}}$ , primer-template binding (annealing) constant (μM^−1^);

The template binding rate *η* is:


(6) \begin{eqnarray*}
\begin{array}{@{}*{1}{c}@{}} {\eta = \frac{{{{c}_b}}}{{{{c}_b} + {{c}_{t,ss}} + 2{{c}_{t,ds}}}}}\\ { \approx \frac{{{{c}_b}}}{{{{c}_b} + 2{{c}_{t,ds}}}}}\\ { = \frac{{{{K}_2}{{c}_p}{{c}_{t,ss}}}}{{{{K}_2}{{c}_p}{{c}_{t,ss}} + 2{{K}_1}c_{t,ss}^2}}}\\ { = \frac{{{{c}_p}}}{{{{c}_p} + 2{{K}_A}{{c}_{t,ss}}}} } \end{array}
\end{eqnarray*}

Assuming that annealing constants are directly proportional to DNA length, i.e.


(7) \begin{eqnarray*}
{{K}_A} = \frac{{{{K}_1}}}{{{{K}_2}}} \propto \frac{{{{S}_a}}}{{{{S}_p}}}
\end{eqnarray*}

Since ${{c}_t} = {{c}_b} + {{c}_{t,ss}} + 2{{c}_{t,ds}}$, substitute *c*_*t*,ss_ with $c_t^\nu $, where *ν* represents the intensity of the impact of real-time amplicon concentration on primer-template binding efficiency, thus,


(8) \begin{eqnarray*}
\eta \approx \frac{{{{c}_p}}}{{{{c}_p} + 2{{K}_A} \cdot c_t^\nu }}
\end{eqnarray*}

(9) \begin{eqnarray*}
\xi = {{\log }_2}\left( {1 + \eta } \right) \approx {{\log }_2}\left( {1 + \frac{{{{c}_p}}}{{{{c}_p} + 2{{K}_A} \cdot c_t^\nu }}} \right)
\end{eqnarray*}

Thus, *η* and *ξ* depend on relative primer-template binding efficiency (${{K}_A} = \frac{{{{K}_1}}}{{{{K}_2}}} \propto \frac{{{{S}_a}}}{{{{S}_p}}}$) and primer-to-amplicon concentration ratio. Primer depletion over time is given by:


(10) \begin{eqnarray*}
{{c}_{p,t}} = {{c}_{p,0}} - \left( {{{c}_t} - {{c}_0}} \right)
\end{eqnarray*}

yielding a time-dependent binding rate:


(11) \begin{eqnarray*}
{{{\boldsymbol{\eta }}}_{\boldsymbol{t}}} = \frac{{{{{\boldsymbol{c}}}_{{\boldsymbol{p}},{\boldsymbol{t}}}}}}{{{{{\boldsymbol{c}}}_{{\boldsymbol{p}},{\boldsymbol{t}}}} + 2{{{\boldsymbol{K}}}_{\boldsymbol{A}}} \cdot {\boldsymbol{c}}_{\boldsymbol{t}}^{\boldsymbol{v}}}}
\end{eqnarray*}

Substituting *η_t_* into Eq. 1 provides the copy number at any time:


(12) \begin{eqnarray*}
{{{\boldsymbol{N}}}_{{\boldsymbol{t}} + \Delta {\boldsymbol{t}}}} = {{\left( {1 + {{{\boldsymbol{\eta }}}_{\boldsymbol{t}}}} \right)}^{\frac{{\Delta {\boldsymbol{t}}}}{{{{{\boldsymbol{T}}}^{\boldsymbol{\theta }}}}}}}{{{\boldsymbol{N}}}_{\boldsymbol{t}}}
\end{eqnarray*}

*N*_*t*_ (copies/μl) and *c*_*t*_ (μM) can be converted via:


(13) \begin{eqnarray*}
{{{\boldsymbol{c}}}_{\boldsymbol{t}}} = \frac{{{{{\boldsymbol{N}}}_{\boldsymbol{t}}}}}{{\boldsymbol{R}}} \times {{10}^{12}}
\end{eqnarray*}

Where *R* is Avogadro’s number, 6.02 × 10^23^ (mol^−1^)

*T* ^θ^ is not directly measurable due to approximations (e.g. *c*_*t*,ss_→${\boldsymbol{c}}_{\boldsymbol{t}}^{\boldsymbol{v}}$, GC-content effects). Similarly, *K*_*A*_ cannot be directly determined, yet its magnitude is expected to be comparable to *S*_*a*_/*S*_*p*_ given their proportionality. Therefore, the parameters (*T*^θ^, *K*_*A*_, and *v*) are obtained by fitting Eq. 12 to amplification curves.

### Poisson-distributed LAMP amplicons predicted by mathematical equivalence

Notably, *S*_*a*_ in the model may represent either the size of the conventional IEA product or the repeating unit size in structured amplicons (e.g. LAMP). Although LAMP generates heterogeneous products, including stem-loop and cauliflower-like structures with multiple loops [16, 17], digestion with restriction endonucleases yields uniform fragments [10, 17]. This consistency arises because all products comprise inverted repeats of the target sequence confined by inner primers (F2/B2).

Mechanistically, Bst DNA polymerase’s strand-displacement activity releases a new primer-binding site upon completing each repeating unit. Consequently, despite structural complexity, LAMP kinetics obey Eq. 12, where *S*_*a*_​ specifically denotes the repeating unit length.

Despite the complexity of LAMP amplification products, the fundamental equivalence between LAMP and simple exponential amplification is revealed through Taylor expansion of the exponential function:


(14) \begin{eqnarray*}
{{2}^{\boldsymbol{x}}} = 1 + \frac{{{\boldsymbol{x}}\ln 2}}{{1!}} + \frac{{{{{\left( {{\boldsymbol{x}}\ln 2} \right)}}^2}}}{{2!}} + \frac{{{{{\left( {{\boldsymbol{x}}\ln 2} \right)}}^3}}}{{3!}} + \ \
\end{eqnarray*}

Substituting ${\boldsymbol{x}} = \frac{{\Delta {\boldsymbol{t}}}}{{{{{\boldsymbol{T}}}^{\boldsymbol{\theta }}}}} = \frac{{{\boldsymbol{t}} - {{{\boldsymbol{t}}}_0}}}{{{{{\boldsymbol{T}}}^{\boldsymbol{\theta }}}}}$ into the equation and multiplying by ${{{\boldsymbol{N}}}_{{{{\boldsymbol{t}}}_0}}}$ gives the amplicon count at time *t*:


(15) \begin{eqnarray*}
&&{\boldsymbol{N}}_{{\boldsymbol{t}}_{0}} {{2}^{\frac{{\Delta {\boldsymbol{t}}}}{{{{{\boldsymbol{T}}}^{\boldsymbol{\theta }}}}}}}\\&=& {\boldsymbol{N}}_{{\boldsymbol{t}}_{0}} \left(1 + \frac{{\frac{{\Delta {\boldsymbol{t}}}}{{{{{\boldsymbol{T}}}^{\boldsymbol{\theta }}}}}\ln 2}}{{1!}} + \frac{{{{{\left( {\frac{{\Delta {\boldsymbol{t}}}}{{{{{\boldsymbol{T}}}^{{\theta }}}}}} \right)}}^2}{{{\left( {\ln 2} \right)}}^2}}}{{2!}} + \ldots + \frac{{\left( {\frac{{\Delta {\boldsymbol{t}}}}{{{{{\boldsymbol{T}}}^{{\theta }}}}}} \right)}^{m}(\ln \,2)^{\boldsymbol{m}}}{{\boldsymbol{m}}!}+ \ldots\right)\\
\end{eqnarray*}

Which can be rewritten as:


(16) \begin{eqnarray*}
{{{\boldsymbol{N}}}_{{{{\boldsymbol{t}}}_0}}}{{2}^{\frac{{\Delta {\boldsymbol{t}}}}{{{{{\boldsymbol{T}}}^{\boldsymbol{\theta }}}}}}} = {{{\boldsymbol{N}}}_{{{{\boldsymbol{t}}}_0}}} \cdot \mathop {\underbrace {{{{\boldsymbol{e}}}^{\frac{{\Delta {{\bf t}} \cdot \ln 2}}{{{{{\boldsymbol{T}}}^{\boldsymbol{\theta }}}}}}}}_{{\boldsymbol{amplification}}\ {\boldsymbol{factor}}}}\limits_{} \cdot \mathop \sum \limits_{{\boldsymbol{m}} = 0}^\infty \underbrace {\mathop {\left[ {{{{\boldsymbol{e}}}^{ - \frac{{\Delta {{\bf t}} \cdot \ln 2}}{{{{{\boldsymbol{T}}}^{\boldsymbol{\theta }}}}}}} \cdot \frac{{{{{\left( {\frac{{\Delta {\boldsymbol{t}} \cdot \ln 2}}{{{{{\boldsymbol{T}}}^{\boldsymbol{\theta }}}}}} \right)}}^{\boldsymbol{m}}}}}{{{\boldsymbol{m}}!}}} \right]}\limits_{} }_{{\boldsymbol{Poisson}}\ {\boldsymbol{distribution}}}\\
\end{eqnarray*}

The left-hand side represents the expression for ideal simple exponential amplification. The right-hand side provides an approximate description of the LAMP amplification pattern, where:

- The zeroth term (${{{\boldsymbol{N}}}_{{{{\boldsymbol{t}}}_0}}} \cdot 1,\ {\boldsymbol{m}} = 0$) corresponds to the contribution of amplicons at *t*_0_.
- The first term (${{{\boldsymbol{N}}}_{{{{\boldsymbol{t}}}_0}}} \cdot \frac{{\frac{{\Delta {\boldsymbol{t}}}}{{{{{\boldsymbol{T}}}^{\boldsymbol{\theta }}}}}\ln 2}}{{1!}},\ {\boldsymbol{m}} = 1$) represents the contribution of products with one repeat unit during $\Delta {\boldsymbol{t}}$.
- The *m*-th term (${{{\boldsymbol{N}}}_{{{{\boldsymbol{t}}}_0}}} \cdot \frac{{{{{( {\frac{{\Delta {\boldsymbol{t}}}}{{{{{\boldsymbol{T}}}^{\boldsymbol{\theta }}}}}} )}}^{\boldsymbol{m}}}{{{( {\ln 2} )}}^{\boldsymbol{m}}}}}{{{\boldsymbol{m}}!}},\ {\boldsymbol{m}} \ge 2$) represents the contribution from products containing *m* repeat units.

This expansion predicts the generation of heterogeneity in LAMP products as a Poisson process. The fractional abundance of products with *m* repeat units is given by:


(17) \begin{eqnarray*}
{\boldsymbol{p}}\left( {\boldsymbol{m}} \right) = {{{\boldsymbol{e}}}^{ - {\boldsymbol{\lambda }}}} \cdot \frac{{{{{\left( {\boldsymbol{\lambda }} \right)}}^{\boldsymbol{m}}}}}{{{\boldsymbol{m}}!}},\ {\boldsymbol{\lambda }} = \frac{{\Delta {\boldsymbol{t}} \cdot \ln 2}}{{{{{\boldsymbol{T}}}^{\boldsymbol{\theta }}}}}
\end{eqnarray*}

The Poisson distribution directly links *∆t* and *T*^θ^ to fragment abundance ratios of LAMP amplification products. The Poisson parameter ${\boldsymbol{\lambda }} = \frac{{\Delta {\boldsymbol{t}} \cdot \ln 2}}{{{{{\boldsymbol{T}}}^{\boldsymbol{\theta }}}}}$ represents the mean number of repeat units added per template during the time interval *∆t*, which is consistent with the amplification mechanism of LAMP (assuming a constant rate of enzyme extension and no spatial hindrance). Thus, LAMP amplification, despite its structural complexity, is mathematically isomorphic to the Taylor expansion of simple exponential growth, preserving its fundamental kinetic signature.

## Materials and methods

### Experimental design

To validate the mathematical model, this study systematically evaluated LAMP as a representative IEA technique (Supplementary Fig. S2 details the workflow). To isolate IEA’s core quantitative mechanism while avoiding confounding factors, we used diluted PCR amplicons as templates. This approach minimizes interference from reaction inhibitors and nontarget nucleic acids, which may trigger nonspecific amplification in complex clinical samples. Target SARS-CoV-2 gene fragments (*N, S*, and *N1*) were first derived from a pseudo-virus via reverse transcription PCR (RT-PCR) and subjected to LAMP amplification across serial dilutions. Crucially, IEA reaction kinetics, defined by the doubling time *T* (Eq. 5) and the composite primer-template binding efficiency parameter *ξ* (Eq. 9), dictate that any variable modulating the core parameters (*S*_*a*_, *S*_*e*_, and *ξ*) directly governs amplification efficiency. Consequently, we investigated four critical determinants: enzyme concentration (primarily altering strand extension rate *S*_*e*_), reaction temperature (simultaneously affecting *S*_*e*_ and binding efficiency *ξ*), amplicon size (governing both *S*_*a*_ and *ξ*), and inhibitors (compromising *S*_*e*_ and *ξ*).

### Template acquirement

A pseudo-SARS-CoV-2 strain (SARS-COV-2-abSMNE) was acquired from Sangon Biotech (Shanghai) as a stock solution (∼10^9^ copies/ml). This enveloped pseudo-virus contains partial SARS-CoV-2 RNA sequences spanning ORF1a/b, S, E, M, and N gene segments (total length: 4030 nt; Supplementary Fig. S3). Viral RNA was extracted using the MagicPure^®^ Viral DNA/RNA Kit (TransGen Biotech, Beijing). Three LAMP primer sets targeting SARS-CoV-2 N and S genes were evaluated (Supplementary Table S1). Target gene fragments were amplified via RT-PCR with TransScript^®^ II Green One-Step qRT-PCR SuperMix (TransGen Biotech) using LAMP outer primers (F3/B3). Amplicons were confirmed by gel electrophoresis, purified by excision at expected sizes, and extracted with the TIANgel Purification Kit (TIANGEN Biotech, Beijing). Purified fragments underwent sequencing to verify specificity before storage at –20°C.

### Simulated quantification of target genes

Amplification products from three target genes were combined to form a stock solution, with the concentration of each gene designated *c*_0,*i*_. Serial ten-fold dilutions of the stock solution generated working solutions at various concentrations. These solutions served as templates in loop-mediated isothermal amplification (LAMP) assays to simulate target gene quantification.

LAMP reactions were performed using Bst 2.0 WarmStart^®^ DNA Polymerase (New England Biolabs, NEB, Ipswich, MA, USA) according to the manufacturer’s standard protocol (Supplementary Table S2). Reactions were conducted on a QuantStudio^™^ 1 Real-Time PCR System (Applied Biosystems, Thermo Fisher Scientific, Waltham, MA, USA), with fluorescence signals automatically acquired every 20 s. Each sample was assayed in triplicate; results are presented as mean values ± standard deviation (error bars).

Consistent with isothermal exponential amplification (IEA) theory, the initial target gene concentration (*c_0,i_*) in the stock solution does not influence the doubling time (*T*) or the slope of the standard curve (Eq. S9). Consequently, normalized concentrations (*c_0_*/*c_0,i_*) were used for data analysis. A plot of the time-to-positive (*t*_*P*_) versus the natural logarithm of the normalized concentration (ln(*c_0_*/*c_0,i_*)) yielded a linear standard curve. Outliers exhibiting significant deviation were excluded prior to linear regression. The doubling time (*T*) was calculated from the slope of this standard curve using Eq. S9.

Amplification curves were also fitted to the model (Eq. 12) to determine all parameters (*T*^θ^, *K*_*A*_, and *ν*). For comparison, quantitative PCR (qPCR) targeting crAssphage CPQ_056 [18] was performed using serial template dilutions, and the corresponding model parameters (${{{\boldsymbol{\eta }}}^{\boldsymbol{\theta }}}$, *K*_*A*_, and *v*) were similarly determined by curve fitting (refer to Supplementary Document S4. Mathematical model for qPCR). MATLAB scripts and example experimental amplification curve data are provided for reference (see Supplementary Materials).

### Effect of enzyme concentration and temperature

Temperature and polymerase concentration are key factors influencing LAMP amplification efficiency. To examine the effect of enzyme concentration, we compared reactions at full [E] and half ([E]/2) concentrations. To assess temperature effects, we conducted LAMP reactions at 63°C, 65°C, and 67°C. Variation in the doubling times (*T* from the standard curve and *T*^θ^ from model fitting) under these conditions reflects changes in overall amplification efficiency. Correspondingly, variations in the kinetic parameters *K*_A_ and *v* indicate alterations in primer-template binding efficiency.

### Inhibitor effect

Residual matrix inhibitors in nucleic acid extracts can interfere with downstream amplification. To assess inhibitor effects, serial 10-fold dilutions of target gene stock solutions were prepared. Subsequently, 10 µl aliquots of each dilution were spiked into 90 µl aliquots of sewage viral nucleic acid (SVNA) extract.

SVNA preparation: Fresh sewage samples were centrifuged (4000 × *g*, 5 min) and the pellet discarded. Viral particles in the supernatant were precipitated overnight at 4°C using polyethylene glycol (PEG 8000, 3.5 g) and NaCl (0.79 g) per 35 ml of supernatant. Following centrifugation (15 000 × *g*, 1 h), the sediment was subjected to nucleic acid extraction using the MagicPure^®^ Viral DNA/RNA Kit (TransGen Biotech, Beijing, China).

For comparison, target gene dilutions were spiked into 5-fold diluted SVNA (dSVNA), expected to exhibit reduced inhibition. Amplification efficiency under both conditions (SVNA versus dSVNA) was evaluated by comparing doubling times (*T*), calculated from the slopes of their respective standard curves.

### Dynamic monitoring of LAMP amplicon size distribution

To evaluate the temporal changes in the size distribution of LAMP amplicons, we repeated the LAMP assay targeting the *N* gene (with *N*_0_ ≈ 10^6^ copies/reaction). To simplify the resulting amplicon size profile, no loop primers were included in the reaction mixture. Individual LAMP reactions were terminated at specific time intervals (every 20 s) by heat inactivation at 85°C for 5 min. The amplification products from each time point were subsequently analyzed by electrophoresis using an Agilent 4200 TapeStation System.

### Relative quantification

Relative abundance (*R*_*a*_) between target genes was calculated for each experimental group by applying Eq. S12 across all data points of the standard curve (comprising at least five serial dilution points). The reported values represent the mean ± standard deviation derived from these multiple independent calculations per condition. To validate the relative quantification approach, we adjusted three target gene fragments to near-equimolar concentrations using a Nano-300 spectrophotometer (Hangzhou Allsheng Instruments, Hangzhou, China) and confirmed via LAMP that *R*_*a*_ approached unity.

To simulate SARS-CoV-2 quantification in sewage, serial dilutions of *N* gene fragments were spiked into SVNA. CrAssphage (an established sewage biomarker [19]) served as the reference target. Both *N* gene and crAssphage (targeting its portal protein gene crAss_pp91; Supplementary Table S1) were quantified by LAMP. *R*_*a*_ (*N* gene/crAssphage) was calculated using Eq. S15.

To verify robustness, spiked SVNA samples underwent 5-fold dilution followed by re-quantification. Consistent *R*_*a*_ values post-dilution would confirm matrix effect resistance, as predicted by IEA theory where $\ln {{{\boldsymbol{R}}}_{\boldsymbol{a}}} \propto \Delta {{{\boldsymbol{t}}}_{\boldsymbol{P}}}$ (Eq. S16; $\Delta {{{\boldsymbol{t}}}_{\boldsymbol{P}}} = {{{\boldsymbol{t}}}_{{\boldsymbol{P}},{\boldsymbol{s}}}} - {{{\boldsymbol{t}}}_{{\boldsymbol{P}},{\boldsymbol{t}}}}$). We visualized ln*R*_*a*_ versus *∆t*_*P*_ across varying kinetic parameters (*T*_s_, *K*_*T*_).

## Results

### Analysis of the IEA kinetic model

We describe IEA kinetics using a recurrence relation for amplicon copy number *N*_*t*_(Eq. 12). The apparent doubling time ${\boldsymbol{T}} = \frac{{{{{\boldsymbol{T}}}^{\boldsymbol{\theta }}}}}{{\boldsymbol{\xi }}} = \frac{{{{{\boldsymbol{S}}}_{\boldsymbol{a}}}}}{{{\boldsymbol{\xi }} \cdot {{{\boldsymbol{S}}}_{\boldsymbol{e}}}}}$ integrates three key factors: enzyme kinetics (*S*_*e*_, DNA polymerase extension rate in bp/min), template geometry (*S*_*a*_​, apparent amplicon size in bp), and annealing energetics (*ξ*, transient primer-template binding efficiency). Crucially, *ξ* (${\boldsymbol{\xi }} \approx {{\log }_2}( {1 + \frac{{{{{\boldsymbol{c}}}_{\boldsymbol{p}}}}}{{{{{\boldsymbol{c}}}_{\boldsymbol{p}}} + 2{{{\boldsymbol{K}}}_{\boldsymbol{A}}} \cdot {\boldsymbol{c}}_{\boldsymbol{t}}^{\boldsymbol{\nu }}}}} )$) depends on primer concentration (*c*_*p*​_), amplicon concentration (*c*_*t*​_), and the annealing constants (*K*_*A*_). Here, ${{{\boldsymbol{K}}}_{\boldsymbol{A}}} = \frac{{{{{\boldsymbol{K}}}_1}}}{{{{{\boldsymbol{K}}}_2}}} \propto \frac{{{{{\boldsymbol{S}}}_{\boldsymbol{a}}}}}{{{{{\boldsymbol{S}}}_{\boldsymbol{p}}}}}$ quantifies competitive template self-annealing (Supplementary Fig. S1C), and the exponent *ν* represents the intensity of the impact of real-time amplicon concentration (*c*_*t*_) on primer-template binding efficiency.

**Figure 1.**
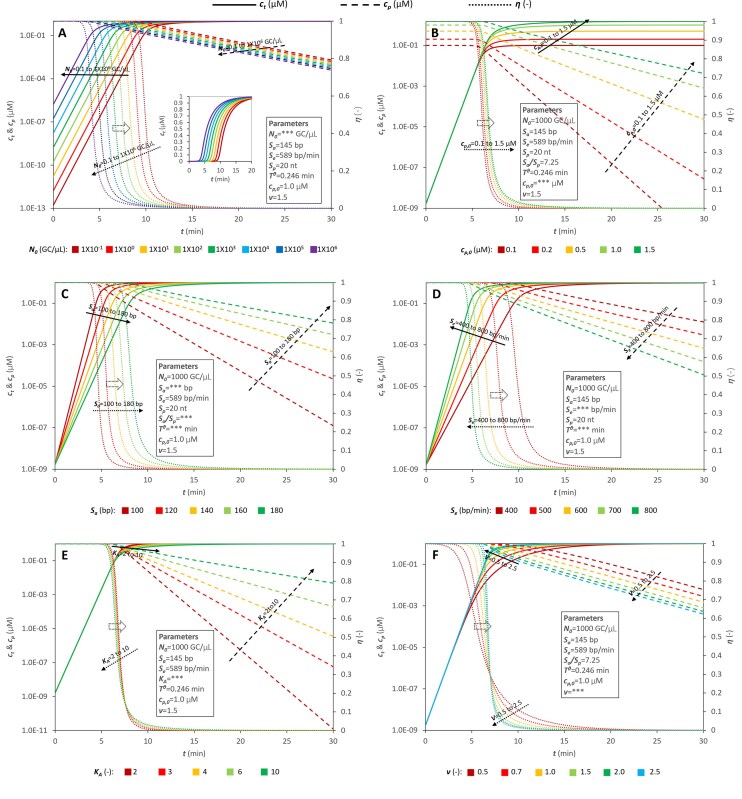
The impact of initial conditions and model parameters on IEA was simulated via model calculation. Initial copy number of target gene *N*_0_ (**A**), the initial concentration of primer *c*_*p*,0_ (**B**), the amplicon size *S*_*a*_ (**C**), the extension rate of DNA polymerase *S*_*e*_ (**D**), the relative binding efficiency between primer and template *K*_A_ (**E**), and the parameter *v* (**F**) were investigated. Solid lines indicate the concentration of the target amplicon at time *t* (*c*_*t*_); dashed lines are real time concentrations of primer (*c*_*p*_); dotted lines exhibit the values of *η* changing over time. The values of *η* refer to the secondary axis, indicating with a hollow dotted arrow in each panel. The embedded diagram in panel (A) gives the amplification curves in linear coordinate system.

Figure 1 presents simulation results using the model. Model parameters are specified in each panel. Under constant reaction conditions (including constant *S*_*e*_), the exponential phase of amplification curves (plotting *c*_*t*_ versus *t*) exhibits a constant slope. Increasing the initial target copy number (*N*_0_) shifts the amplification curve leftward along the time axis (solid lines in Fig. 1A). During the initial amplification phase (*c*_*p*_ >> *c*_*t*_), *η* remains approximately constant (close to 1). As amplification progresses, the amplicon concentration (*c*_*t*_) increases exponentially (solid lines, Fig. 1), depleting primers (*c*_*p*_, dashed lines, Fig. 1). In the later stage of the reaction, the value of *η* decreases rapidly (dotted lines, Fig. 1), and the amplification deviates from exponential growth. The initial primer concentration (*c*_*p*,0_) does not affect the doubling time or the slope of the exponential phase, but it determines the final amplicon concentration (Fig. 1B). Both the amplicon size (*S*_*a*_) and the polymerase extension rate (*S*_*e*_) influence the ideal doubling time (*T^θ^*). Smaller *S*_*a*_ and higher *S*_*e*_ steepen the amplification curves (solid lines, Fig. 1C and D, respectively). A smaller *K*_A_ value (indicating higher primer-template binding efficiency) extends exponential phase; however, within the investigated range (*K*_A_ = 2–10), its impact is modest (Fig. 1E). A higher value of the empirical parameter *ν* (>1.5) delays the transition out of exponential growth (Fig. 1F).

### Validation: enzyme, temperature, and inhibitor effects

#### Doubling time affected by enzyme concentration

**Figure 2.**
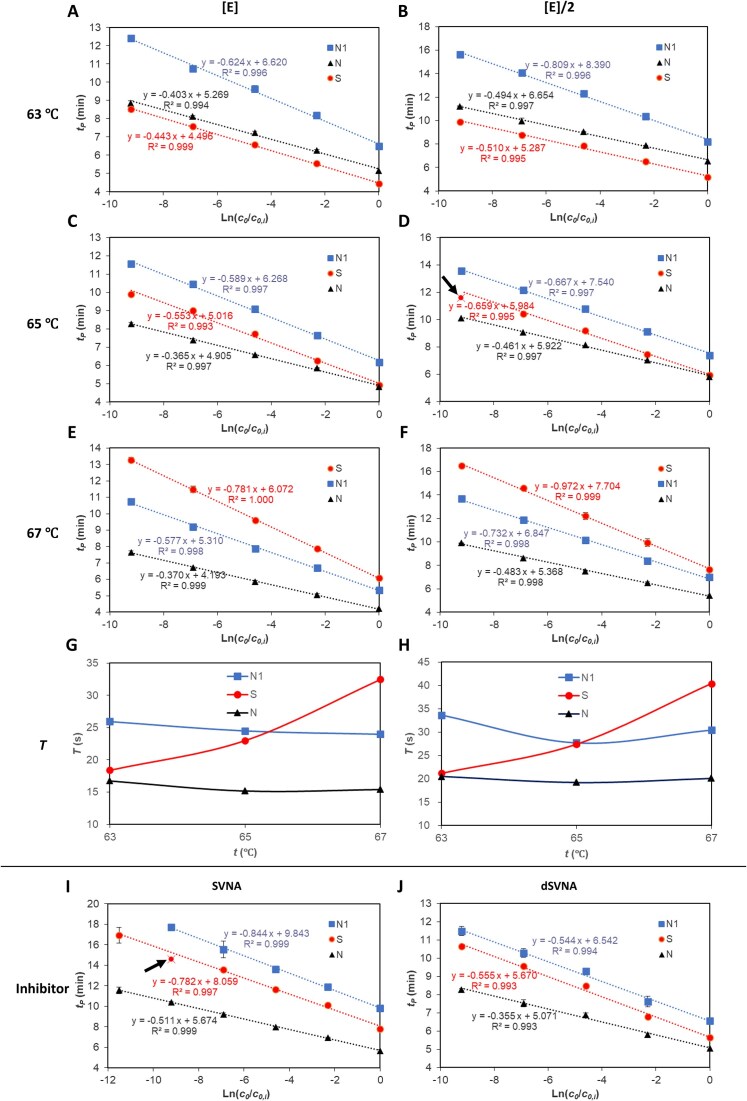
The effects of temperature (63°C, 65°C, or 67°C), enzyme concentration ([E] or [E]/2) and matrix inhibitors on the efficiency of LAMP. Panels (**A**–**F**) show amplification standard curves for three genes (*N, N1*, and *S*) across three temperatures (63°C, 65°C, and 67°C) and two polymerase concentrations ([E] and [E]/2). Specifically, 63°C: A [E], B ([E]/2); 65°C: C [E], D ([E]/2); 67°C: E [E], F ([E]/2). Panels (**G**) and (**H**) demonstrate the temperature dependence of doubling time at enzyme concentrations [E] and [E]/2, respectively. Changing the temperature not only affected the doubling time of each gene (varying the slope of standard curves under different temperatures) but also altered the relative position of different genes’ standard curves in the coordinate system. Decreasing enzyme concentration increases the doubling time (comparing panel G against H) but did not alter the relative position of different genes’ standard curves (comparing standard curves in panel A against B, C against D, and E against F, respectively). Matrix inhibitor effects were accessed by spiking targets into sewage virus nucleic acid extract (SVNA, panel **I**) and 5-fold diluted SVNA (dSVNA, panel J). Obviously deviated data points are excluded as outlier when doing linear regression and are marked with arrows in panel (D) and panel (I).

Halving polymerase concentration increased the apparent doubling time *T* by 13.2%–30.5% (Table 1). According to the model (${\boldsymbol{T}} = \frac{{{{{\boldsymbol{T}}}^{\boldsymbol{\theta }}}}}{{\boldsymbol{\xi }}} = \frac{{{{{\boldsymbol{S}}}_{\boldsymbol{a}}}}}{{{\boldsymbol{\xi }} \cdot {{{\boldsymbol{S}}}_{\boldsymbol{e}}}}}$), reducing polymerase concentration primarily decreases the extension rate *S*_*e*_, thereby increasing *T*. Crucially, since changing polymerase concentration affects *S*_*e*_ equally for different target genes, the ratio of doubling time (${{K}_T} = \frac{{{{T}_s}}}{{{{T}_t}}}$, Eq. S14) between target genes should be largely independent of polymerase concentration. Consistent with this prediction, reducing enzyme concentration did not significantly alert the *K*_*T*_ values between different target genes (Table 1, *P*= 0.12, paired *t*-test). Consequently, under constant temperature conditions, the relative positions of the quantitative standard curves for the three target genes remained stable across different polymerase concentrations (Fig. 2A–F).

**Table 1.** Estimated model parameters at different experimental conditions with Eq. S8 and Eq. S12 according to Fig. 2

Temperature (°C)	Experimental conditions	*T* (min)			*K*_*T*_		*R*_a_ ^d^	
		*N*(145 bp)^b^	*N1*(175 bp)^b^	*S*(151 bp)^b^	*N*/*N1*	*N*/*S*	*N*/*N1*	*N*/*S*
63	[E]	0.279	0.433	0.307	0.645	0.910	0.087 ± 0.025	0.054 ± 0.009
	[E]/2	0.342	0.561	0.353	0.610	0.968	0.045 ± 0.010	0.045 ± 0.007
65	[E]	0.253	0.408	0.384	0.620	0.660	0.063 ± 0.016	0.013 ± 0.005
	[E]/2	0.320	0.462	0.457	0.692	0.700	0.218 ± 0.014	0.021 ± 0.005
	SVNA^a^	0.354	0.585	0.542	0.605	0.653	1.749 ± 0.186	0.416 ± 0.150
	dSVNA^a^	0.246	0.377	0.385	0.653	0.641	0.106 ± 0.009	0.018 ± 0.001
67	[E]	0.256	0.400	0.541	0.641	0.474	0.119 ± 0.008	0.029 ± 0.003
	[E]/2	0.334	0.508	0.674	0.659	0.497	0.171 ± 0.027	0.042 ± 0.012
$\ S_e^*{\mathrm{\ }}$ (bp/min)^c^		589.4(65°C)	464.2(65°C)	491.9(63°C)				

a SVNA: sewage viral nucleic acid; dSVNA: diluted sewage viral nucleic acid.

b The numbers in the blankets indicate the apparent sizes of amplification products (*S*_a_) for three genes.

c The apparent DNA-strand extension rate *S*_e_* was calculated with the formula $S_e^* = \frac{{{{S}_a}}}{{{{T}_{min}}}}$, where *T*_min_ is the minimum doubling time for each target gene fragment.

d Data are presented as mean ± standard deviation (*n* = 5), where *n* represents the number of serial dilution points used for independent calculation of *R*_*a*_ via Eq. S12.

Note: The two-tailed paired *t*-test showed that a decrease in enzyme concentration (*P *= 4.930 × 10^−5^) and the presence of inhibitors (*P *= 0.032) significantly increased the doubling time of the reaction, while there was no significant trend in the effect of temperature on the doubling time (*P *> 0.1). To investigate the effect of enzyme concentration, experiments at 63°C and 67°C were performed using the same dilution series of the target nucleic acid at each temperature. For the SVNA experimental group, the concentrations of the three genes were adjusted to approximately equal levels based on quantification with a Nano-300 micro-spectrophotometer (Allsheng Instruments Co., Ltd., Hangzhou, China).

#### Doubling time affected by temperature

Temperature influences both the DNA polymerase extension rate (*S*_*e*_) and the primer-template binding efficiency (*ξ*). To simplify analysis, we define a composite parameter, the apparent strand extension rate $S_e^* = \xi \cdot {{S}_e}$, which integrates intrinsic polymerase kinetics (*S*_*e*_) and annealing energetics (*ξ*). While elevated temperatures accelerate reaction kinetics, enhanced molecular thermal motion reduces primer-template binding efficiency. Furthermore, excessively high temperatures inactivate the polymerase. Consequently, an optimal reaction temperature exists to minimize the doubling time (*T*). This optimum varies across target gene fragments due to differences in primer annealing temperatures.

Unlike enzyme concentration, temperature exerts complex effects on reaction kinetics and can significantly alter the doubling time ratio *K*_*T*_. Experimentally, *K*_*T, N/S*_ varied substantially with temperature (Table 1), whereas *K*_*T, N*/*N1*_ remained stable. This divergence arose because the temperature-dependent change in *T* for the *S* gene differed markedly from the *N* and *N1* genes (Fig. 2G and H; Table 1). Specifically, for *N* and *N1, T* changed minimally with temperature, with an optimum at 67°C; for *S, T* increased significantly with temperature, exhibiting an optimum at 63°C.

Collectively, temperature-dependent variation in *K*_*T*_ reflects shifts in *ξ* due to altered annealing energetics.

#### Enzyme activity affected by sewage inhibitors

To assess the effect of residual matrix inhibitors, we spiked target gene fragments into SVNA extract. Components of the sewage matrix significantly inhibited IEA, increasing the apparent doubling time (*T*) by 40.8%–55.2% (Fig. 2I, and Tables 1 and 2). However, dilution of the SVNA extract (dSVNA) restored reaction efficiency (*T*) to near-baseline levels (Fig. 2J, and Tables 1 and 2).

**Table 2.** Model parameters determined via fitting the model (Eq. 12) with amplification curves (Fig. 3)

Temperature (°C)	Experimental conditions	*T* ^θ^ (min)			*N_0,m_* (copies/μl)			*K_A_*			*v*			*MSE* × 10^4^ (mean squared error)		
		*N*(145 bp)	*N1*(175 bp)	*S*(151 bp)	*N*	*N1*	*S*	*N*	*N1*	*S*	*N*	*N1*	*S*	*N*	*N1*	*S*
63	[E]	0.339	0.459	0.298	5.47e5	2.99e6	2.45e5	5.962	3.120	5.668	1.398	0.630	1.227	6.732	7.371	4.435
	[E]/2	0.394	0.591	0.338	1.15e5	2.58e6	1.48e5	6.419	3.429	6.219	1.296	0.595	1.171	5.326	1.787	2.269
65	[E]	0.294	0.445	0.385	2.64e5	4.53e6	2.33e6	7.040	2.955	4.617	1.579	0.755	1.561	14.00	52.00	3.944
	[E]/2	0.358	0.538	0.432	2.48e5	2.97e6	9.87e5	8.365	3.238	6.009	2.072	1.025	1.580	3.921	4.934	4.781
	SVNA	0.410	0.681	0.612	1.72e6	3.31e6	3.65e6	6.331	2.435	3.304	1.792	0.693	1.638	7.100	6.934	5.199
	dSVNA	0.274	0.421	0.396	5.39e4	8.03e5	8.29e5	6.077	2.499	3.810	1.854	1.079	1.513	8.060	65.00	4.770
67	[E]	0.270	0.448	0.512	4.64e5	1.09e7	3.38e6	5.463	2.593	3.240	1.812	1.077	1.281	3.533	5.251	1.996
	[E]/2	0.310	0.534	0.678	4.16e4	3.21e6	5.21e6	7.821	3.561	3.910	1.647	1.077	1.292	3.315	3.260	2.440
*S_e_* (bp/min)		537(67°C)	415(65°C)	506(63°C)												

*N_0,m_* denotes the predicted initial copy number from model fitting. This value does not represent the true absolute quantity, as it inherently underestimates due to factors discussed in the main text.

Note: The model parameter values under the optimal reaction conditions for each gene fragment are displayed in bold.

This inhibition primarily reduces the DNA polymerase extension rate (*S_e_*), thereby increasing *T*. Critically, exponential amplification magnifies such effects, necessitating caution when quantifying inhibitor-containing samples using standard curves generated in inhibitor-free systems, as significant quantification bias may occur.

Crucially, inhibitors reduce *S_e_* uniformly across target genes, analogous to decreasing enzyme concentration. As expected, sewage matrix inhibitors did not significantly alter *K_T_* values (*K_T_* variation < 10% between SVNA and dSVNA; Table 1). This stability provides a theoretical foundation for reliable relative quantification of target genes in complex matrices.

#### Model parameter estimation by amplification curve fitting

**Figure 3.**
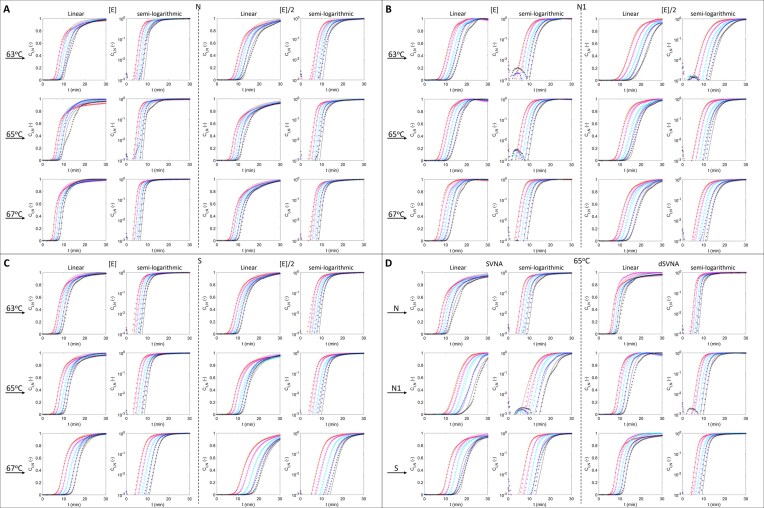
Experimental (star markers) and simulated (solid lines) LAMP amplification curves. LAMP reactions targeting gene fragments *N* (**A**), *N1* (**B**), and S (**C**) were performed across three different temperatures (63°C, 65°C, and 67°C) and two polymerase concentrations ([E], [E]/2). Inhibitor effects were accessed by spiking targets into sewage virus nucleic acid extract (SVNA) and diluted SVNA (dSVNA) (**D**). All amplification curves are displayed in linear and semi-logarithmic formats.

Experimental and simulated amplification curves are presented in Fig. 3, with corresponding model parameters summarized in Table 2. The high concordance between doubling times derived from curve fitting and those calculated from standard curve slopes (refer to Supplementary Document S2. IEA quantification via standard curves) validates the model (Supplementary Fig. S5). Elevated enzyme concentration significantly enhanced primer-template binding efficiency, as indicated by reduced *K_A_* values (Table 2; *P* = 5.88 × 10⁻^4^, paired *t*-test). In contrast, parameter *ν* exhibited no significant correlation with enzyme concentration.

Primer sets and reaction conditions exhibiting shorter doubling times and higher *ν* values (typically > 1.0) enable accelerated quantitative detection. Shorter doubling times correspond to steeper exponential-phase slopes and reduced spacing between serial dilution curves, thereby reducing time-to-detection (Fig. 3). The model facilitates optimal primer selection and reaction optimization. Among three gene-specific primer sets, primer set *N* achieved the shortest doubling time at 67°C, and primer set *S* yielded the shortest doubling time at 63°C. Both generated detectable signals within 10 min under their respective optimal temperatures (Fig. 3).

Under optimized conditions, *ν* consistently exceeded 1.0, indicating delayed departure from exponential kinetics. Given the minimal impact of *K_A_* on amplification profiles (Fig. 1E), it holds the lowest priority for optimization. Furthermore, kinetic fitting results indicate that the threshold for time-to-positive (*t_P_*) determination must be set ≤10% of the plateau fluorescence value.

### LAMP as a Taylor series expansion of exponential growth

The kinetics of LAMP, which generates complex stem-loop DNA products, were mathematically demonstrated to be equivalent to simple exponential amplification (Eq. 16). This equivalence justifies the use of Eq. 12 to model LAMP kinetics despite the heterogeneity of amplicons.

**Figure 4.**
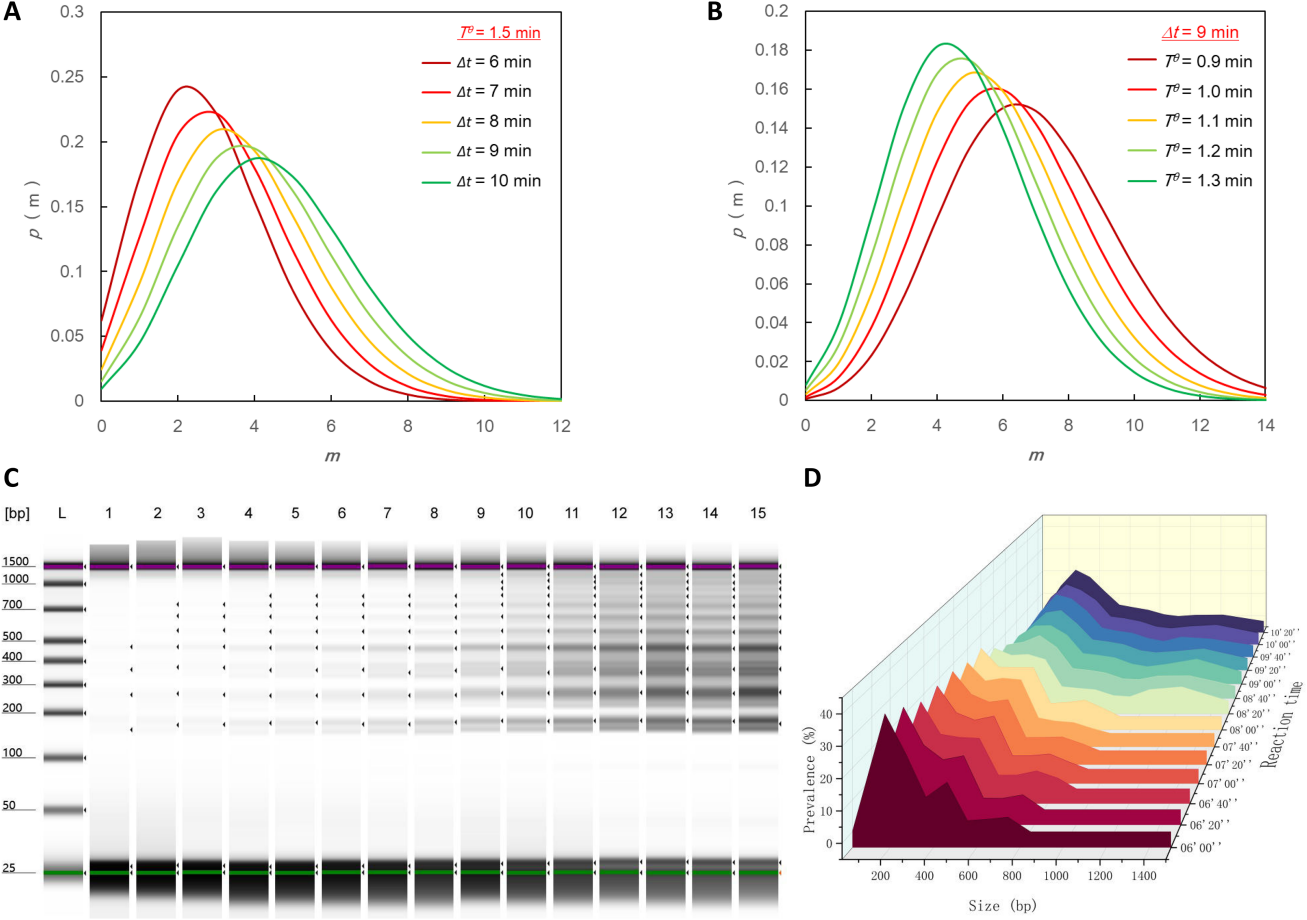
The size distribution of LAMP amplification products follows a Poisson distribution, ($p( m ) = {{e}^{ - \lambda }} \cdot \frac{{{{{( \lambda )}}^m}}}{{m!}},\ \lambda = \frac{{\Delta t \cdot \ln 2}}{{{{T}^\theta }}}$). The parameter *λ* is modulated by the amplification time (*∆t*) and the doubling time (*T^θ^*). Panel (**A**) illustrates how the size distribution varies with amplification time (*∆t*) at a fixed doubling time (*T^θ^*). Conversely, panel (**B**) demonstrates the size distribution variation resulting from differing doubling times (*T^θ^*) at a constant amplification time (*∆t*). Panel (**C**) shows electrophoretic profiles of N gene LAMP products from reactions terminated at time points from 5′40″ (sample 1) to 10′20″ (sample 15), at 20-s intervals. Panel (**D**) displays the corresponding amplicon size distribution for samples collected between 6′00″ and 10′20″ after reaction initiation.

The mathematical framework predicts that the relative abundance of LAMP amplification fragments follows a Poisson distribution, directly linking *∆t* and *T^θ^* to product composition ratios. At the initial amplification stage (*∆t* → 0), the products predominantly consist of small fragments with few repeating units (low *m*). As amplification time increases (*∆t* ↑) or the doubling time decreases (*T^θ^* ↓), the distribution exhibits two key trends: (i) The mean size (*m*) increases linearly with *λ*; (ii) The dispersion of sizes (variance σ² = *λ*) widens monotonically. This results in a shift of the distribution peak toward larger *m* and an increase in size heterogeneity, consistent with a Poisson process. As *m* increases beyond *λ*, the fractional abundance $p( m ) = {{e}^{ - \lambda }} \cdot \frac{{{{{( \lambda )}}^m}}}{{m!}},\ \lambda = \frac{{\Delta t \cdot \ln 2}}{{{{T}^\theta }}}$ decays super-exponentially. This rapid convergence toward zero implies that amplification products with large *m* (i.e. *m*≫*λ*) contribute negligibly to the overall distribution (Fig. 4A and B). Electrophoretic monitoring of the size distribution of LAMP amplification products at different time points confirmed the model predictions. Although electrophoresis could only capture the changes in product size distribution during the late exponential amplification phase, the results demonstrated that as the reaction proceeded, the product size distribution shifted toward larger sizes while gradually broadening (Fig. 4C and D).

### Relative quantification in wastewater

The stability of *K_T_* against global variations in polymerase activity and nonspecific inhibition, as established in the previous sections, provides the theoretical foundation for robust relative quantification. We therefore derived a formula for the relative abundance (*R_a_*) of target (*t*) versus reference (*s*):


(18) \begin{eqnarray*}
{{R}_a} = \frac{{{{c}_{0,t}}}}{{{{c}_{0,s}}}} = {{K}_S} \cdot {{2}^{\frac{{{{t}_{P,s}} - {{K}_T}{{t}_{P,t}}}}{{{{T}_s}}}}}
\end{eqnarray*}

where ${{K}_S} = {{S}_{a,S}}/{{S}_{a,t}}$ and ${{K}_T} = {{T}_s}/{{T}_t}$. The detailed derivation can be found in Supplementary Document S3. Relative quantification.

Our LAMP-derived relative abundance (*R_a_*) values showed high consistency across the multiple dilution points of each standard curve (Table 1), despite the inherent limitations in quantification accuracy common to exponential amplification [20]. The low standard deviations reflect the robustness of these independent calculations. For instance, *R_a_* values were highly consistent across experimental groups that used the same template mixture but varied in reaction conditions (experiments at 63°C and 67°C). Additionally, in the specific sample with deliberately equimolar gene fragments (the SVNA experimental group), the mean *R_a_* approached unity as expected. This high consistency, achieved through a dilution-based statistical approach, validates the predictive power of our kinetic model and demonstrates the feasibility of robust IEA-based relative quantification.

Supplementary Fig. S6 demonstrates mimic relative quantification of SARS-CoV-2 *N* gene in sewage nucleic acid. Standard curves for crAssphage pp99/*N* and *N* genes were constructed using serial dilutions of outer-primer PCR/RT-PCR amplicons (Supplementary Fig. S6A). Key model parameters (*A, T, K_T_*) are summarized in Supplementary Table S3. Serial 10-fold dilutions of SARS-CoV-2 *N* gene fragment were spiked into SVNA and quantified alongside the endogenous sewage biomarker (crAssphage) via LAMP (Supplementary Fig. S6B). To assess robustness, spiked SVNA samples were further diluted (dSVNA) and re-quantified (Supplementary Fig. S6C).

Crucially, *K_T_* remained stable under constant temperature and was minimally affected by polymerase activity or inhibitors (per Sections 4.2.1 and 4.2.3). Thus, *R_a_* values (calculated via Eq. S15) for *N* gene fragment in SVNA and dSVNA samples consistently reflected the dilution ratios (*c_0_*/*c_0,i_*), with deviations < 35% (Dev_*R_a_*%, Supplementary Table S3). This stability enables reliable viral load estimation in complex matrices.

## Discussion

### Positioning our model among existing quantitative frameworks

Current modeling strategies for IEA kinetics fall into two distinct categories, each with inherent limitations that our universal framework aims to address. On one end of the spectrum are empirical, phenomenological models. A common approach is to employ logistic functions to describe IEA kinetics. The foundational work in this area established the use of a generalized logistic function (Richard’s curve) to fit LAMP amplification data and extract a quantitative “time-to-positive” (*T_P_*) [13]. This approach was later refined for simplicity. A three-parameter logistic model was successfully applied to model both LAMP and RPA kinetics [14, 15, 21], demonstrating the broader utility of sigmoidal fits across different IEA techniques. Most recently, this paradigm was extended to endpoint quantification, where a four-parameter logistic fit applied to RPA trajectories enabled the derivation of a normalized endpoint intensity (NEI) for quantitative analysis [22]. While these functions are valuable for post-hoc curve fitting and defining empirical metrics, their parameters are primarily mathematical fitting constants with limited direct physical interpretation, offering little insight into the underlying reaction mechanics or guidance for optimization.

On the opposite end lies detailed, mechanism-specific kinetic modeling, as exemplified by the work of Moody *et al.* for RPA [23]. Their model comprises a system of differential equations that meticulously describe specific biochemical steps, including recombinase-primer binding and strand displacement. While such models provide profound mechanistic insight into a particular technique, their complexity (involving numerous hard-to-measure rate constants) and specificity limit their transferability and practical utility for cross-technique comparison and design.

The broad applicability of a single class of empirical function (the logistic function) across IEA methods suggests a fundamental kinetic commonality. Our universal kinetic model captures this commonality by establishing its physical basis, bridging the gap between purely empirical and overly complex mechanistic approaches. By abstracting the core physics governing all IEA techniques, our model not only recapitulates the entire amplification trajectory but also mechanistically accounts for growth deceleration and plateau through the dynamic template’s primer-binding rate, *η_t_* (Eq. 11). As amplification proceeds and amplicon concentration (*c_t_*) increases dramatically, the value of *η_t_* decreases due to its dependence on $c_t^\nu $ (Eq. 8). This decay in *η_t_* intrinsically captures the collective deceleration of the reaction, which includes contributions from primer depletion, product inhibition, and the effective saturation of enzyme activity when the number of templates vastly exceeds the available polymerase molecules. Thus, while not explicitly modeled as a Michaelis–Menten term, the saturation effect is a significant component of the reaction deceleration and plateau described by our model.

Crucially, the parameters in our model, the intrinsic polymerase speed (*S_e_*), amplicon size (*S_a_*), and the annealing efficiency (*ξ*), possess clear physical interpretations. This transforms the model from a curve-fitting tool into a predictive framework for optimizing reaction design and diagnosing performance bottlenecks across diverse IEA methodologies, from LAMP to RPA and beyond.

### Elucidating the quantitative limitations of IEA versus qPCR

Our kinetic model not only captures IEA behavior but also starkly reveals its fundamental quantitative limitations when compared to qPCR. A direct comparison is illustrative: while our qPCR model achieved near-perfect fits across a wide dynamic range (10^2^ to 10^7^ copies/μl; Supplementary Fig. S8, Supplementary Table S4), the LAMP model showed discernible deviations, particularly at low concentrations (Fig. 3). This empirical observation points to a deeper divergence in robustness.

The root of this disparity lies in the core architectural difference between the techniques. qPCR’s thermal cycling acts as a built-in error-correction mechanism. Each high-temperature denaturation step resets the reaction, confining the impact of minor thermodynamic fluctuations (e.g. in temperature or reagent concentration) to a single cycle. This prevents error accumulation, granting the cycle threshold (*C_T_*) high inter-run reproducibility and underpinning qPCR’s broad linear dynamic range. In contrast, IEA is a continuous process. Any perturbation to the instantaneous doubling time (*T*) is amplified throughout the entire exponential phase. This accumulated error manifests as greater variance in the time-to-positive (*t_P_*) and is quantitatively reflected in the higher mean squared error (MSE) of our LAMP fits compared to qPCR (compare Table 2 and Supplementary Table S4). The elevated MSE quantitatively captures the extent of deviation from the ideal exponential growth model—the very theoretical foundation that enables quantification in these techniques.

This continuous architecture also exacerbates challenges at low template concentrations. The intrinsic stochasticity of strand separation and primer binding under isothermal conditions becomes significant. Furthermore, the constant reaction temperature and unfavorable primer-to-template ratio promote competing side reactions, such as primer-dimer formation and nonspecific amplification. These nonideal pathways consume reagents and generate background signal, directly competing with the specific amplification kinetics described by our model. In qPCR, cyclic denaturation ensures deterministic strand separation, and temperature-cycled annealing promotes highly specific primer binding, largely suppressing these issues.

A telling signature of IEA’s stochastic start-up is the systematic underestimation of the initial target concentration (*N_0,m_* in Table 2) by our model. This occurs because an unmodeled, variable “start-up time”—required for processes like hot-start polymerase activation or initial dumbbell structure formation in LAMP—shifts the entire amplification curve. The fitting algorithm compensates for this rightward shift by underestimating *N_0_* to match the curve’s timing and shape. This effect is absent in qPCR due to its deterministic, cycle-reset nature.

In summary, the quantitative performance of IEA is constrained by a combination of factors that are intrinsically mitigated in qPCR: the accumulation of kinetic errors during continuous amplification, and the increasing influence of stochastic initiation and side reactions at low target concentrations. While often negligible at high template loads, these effects dominate near the limit of detection, explaining the characteristically narrower dynamic range and higher lower limit of quantification (LLOQ) of IEA techniques like LAMP compared to qPCR.

### Optimization strategies

As the primary parameter governing IEA reaction efficiency, doubling time (*T*) is predominantly determined by three factors: the DNA-strand extension rate (*S_e_*), the apparent amplicon size (*S_a_*), and the primer-template binding efficiency (*ξ*). This relationship yields a physically meaningful expression for IEA doubling time, establishing a theoretical framework for optimizing IEA methodologies. Reducing *T* enhances detection efficiency and accelerates result generation.

Minimize *S_a_*: Reducing amplicon size (*S_a_*) is the most effective strategy to shorten doubling time and improve amplification efficiency (Fig. 2B). For instance, the reported ultrarapid SARS-CoV-2 detection via IEA leverages an exceptionally short (17 bp) amplicon [24]. Unlike other IEA techniques, LAMP generates a heterogeneous mixture of DNA products with stem-loop or cauliflower-like structures containing multiple inverted repeats of the target sequence [16, 17]. Incorporating auxiliary primers effectively minimizes the size of these repeating units, thereby enhancing reaction efficiency [25, 26]. Furthermore, smaller amplicons reduce the value of *K_A_* (Eq. 7), improve primer-template binding efficiency *ξ* (Eq. 9), and expedite reaction endpoint attainment (Fig. 1E).

Enhance *S_e_*: The apparent strand extension rate ($S_e^*$) can be derived from the apparent amplicon size (Table 1, bottom row). At 65°C, Bst DNA polymerase exhibits an $S_e^*$ of 589 bp/min. Although lacking 3′→5′ exonuclease activity, Bst polymerase possesses strand-displacing capability. For comparison, Taq DNA polymerase has a standard extension rate of ∼1000 bp/min, while engineered polymerases can achieve rates of 2000–4000 bp/min (https://www.neb.cn/applications/dna-amplification-pcr-and-qpcr/specialty-pcr/fast-pcr). Thus, significant potential exists to enhance *S*_e_ through polymerase engineering.

Maximize *ξ*: Elevated reaction temperatures and rational primer design improve annealing specificity. LAMP exhibits the shortest doubling time (0.30 min or 18 s) among major IEA techniques, as summarized in Supplementary Table S5. Higher operating temperatures generally increase efficiency by accelerating kinetics and suppressing nonspecific amplification; studies confirm that appropriate temperature increases reduce detection time [27]. However, excessively high temperatures may compromise primer annealing or polymerase activity. Reaction efficiency is also critically dependent on primer set design. Optimized primers enhance LAMP sensitivity and broaden its linear dynamic range (reported to span 9 orders of magnitude [10]). Notably, different primer sets targeting distinct segments of the same gene (e.g. *N* vs. *N1* in this study) can exhibit significant efficiency differences (*P* = 4.027 × 10^−5^, paired *t*-test on their *T* values). Primer performance is influenced not only by sequence-specific binding efficiency but also by target sequence characteristics; GC-content and propensity for secondary structure formation impact IEA efficiency. Consequently, $S_e^*$ serves as a composite parameter reflecting enzyme kinetics, primer binding, and target sequence properties, indirectly indicating primer design quality. Through coordinated optimization of polymerase performance and primer design, IEA can potentially yield results within minutes.

Finally, nonspecific amplification must be mitigated during IEA quantification. Standard protocols should employ hot-start polymerase in pre-mixed reagents prepared at low temperatures.

### A universal framework for IEA optimization

The model presented here, $T = \frac{{{{S}_a}}}{{\xi \cdot {{S}_e}}}$, transcends the specifics of LAMP and offers a universal language for comparing and optimizing all IEA techniques. It posits that the efficiency of any IEA method is ultimately governed by the interplay of three fundamental physical parameters: enzyme kinetics (*S_e_*), template geometry (*S_a_*​), and annealing energetics (*ξ*). This framework demystifies why techniques employing short amplicons (e.g. ultrarapid assays [24]) achieve remarkably short doubling times, and provides a clear directive for future polymerase engineering. While auxiliary mechanisms (e.g. strand displacement in LAMP, recombinase invasion in RPA, helicase unwinding in HDA) differ, their kinetic impact is unified through modulation of the effective extension rate (*S_e_*) and binding efficiency (*ξ*). Consequently, the optimization of seemingly disparate IEA methods is reduced to a common set of challenges: maximizing *S_e_*​, minimizing *S_a_*​, and fine-tuning *ξ*. This shift from empirical tuning to principle-driven design, grounded in measurable physical parameters, provides a powerful blueprint for accelerating the development of next-generation IEA diagnostics.

### Future applications

While this study validates the model within the LAMP system, the universal nature of the derived physical principles ($T = \frac{{{{S}_a}}}{{\xi \cdot {{S}_e}}}$) suggests its broad applicability. Future work to derive technique-specific *T^θ^* and experimentally validate the framework across other IEA methods (e.g. RPA and HDA) and using polymerases with distinct extension rates would be a valuable extension of this research.

The successful application of our model for relative quantification in inhibitor-rich wastewater (deviation < 35%) not only demonstrates its practical utility but also validates its core prediction: that the doubling time ratio (*K_T_*) is an intrinsic property resilient to matrix effects. This confirms that the model captures the essential physics of IEA, enabling robust quantification in inhibitor-rich environments without the need for empirical calibration. The relative quantification strategy presented in this study partially mitigates the inherent limitations of IEA for quantitative applications and enables high-throughput POC testing. Theoretical analyses demonstrate that the amplification time difference between target and reference genes (*t_P,s_*–*t_P,t_*) is proportional to the logarithm of their initial concentration ratio (ln*R_a_*) (Supplementary Fig. S9). This relationship closely parallels the comparative *C_T_* method used in qPCR [28]. Consequently, target nucleic acid quantification via IEA can be achieved using a miniaturized portable device integrating temperature control, signal detection, and timing functions.

For absolute quantification, partitioning-based digital IEA (dIEA) platforms utilizing microfluidic technology represent a significant and powerful future direction [27, 29, 30]. Analogous to the way digital PCR has complemented quantitative PCR, dIEA leverages Poisson statistics to count individual amplification events from a partitioned sample, thereby providing absolute quantification without a standard curve. This approach is exemplified by recent advances such as digital LAMP (dLAMP) with high-resolution melt (HRM) analysis [31], which achieves single-molecule sensitivity and, crucially, differentiates specific amplification from nonspecific background—a major challenge in IEA quantification. While addressing nonspecific amplification requires specific experimental strategies like HRM, our universal kinetic framework, defining efficiency through the parameters *S_e_, S_a_*, and *ξ*, provides a rational basis for optimizing such dIEA assays. By guiding the selection of polymerases (for high *S_e_*), primer sets (for minimal effective *S_a_* and optimal *ξ*), and reaction conditions, the model can help maximize the number of partitions that undergo efficient and rapid amplification, thereby improving the accuracy, speed, and detection limit of digital quantification and moving the design of these next-generation assays beyond trial-and-error.

The translation of IEA into regulated quantitative POC diagnostics must also contend with practical challenges, including the mitigation of false positives and navigating a regulatory landscape currently defined by qPCR. The limitations discussed here such as nonspecific amplification and higher LLOQ directly impact diagnostic sensitivity and specificity. Our kinetic framework, by quantifying the determinants of amplification efficiency (*S_e_, S_a_*, and *ξ*), provides a rational path forward. It guides the principled optimization of reaction components and conditions to minimize nonspecific events (e.g. by maximizing *ξ* through primer design and temperature selection) and improve quantitative robustness. This is exemplified by our LAMP model fitting, which identified the optimal reaction conditions for each gene target. The corresponding model parameters achieving the highest fidelity are highlighted in Table 2. By moving assay design from empirical tuning to physics-driven engineering, this model serves as a foundational tool for developing next-generation IEA tests that are not only rapid and portable but also demonstrate the reliability required for clinical approval and widespread POC adoption.

## Supplementary Material

gkaf1378_Supplemental_Files

## Data Availability

All data is contained within the manuscript and/or supplementary files.
