# Supplementary material for: A universal kinetic framework for quantitative isothermal amplification governed by polymerase speed, amplicon size, and binding efficiency
Source: Nucleic Acids Res. 2026 Jan 6;54(1):gkaf1378. doi: 10.1093/nar/gkaf1378 (PMC12774662; doi:10.1093/nar/gkaf1378)
Supplement: gkaf1378_Supplemental_Files [file gkaf1378_supplemental_files.zip › IEA-SI-NAR-R1-correction.pdf]

SUPPLEMENTARY INFORMATION

A universal kinetic framework for quantitative isothermal amplification governed by polymerase speed, amplicon size, and binding efficiency

Langjun Tang, Zhenyu Guo, Jinyong Wu, Yonghong Li*, Kun Yang*

Department of Pharmaceutical & Biological Engineering, School of Chemical Engineering, Sichuan University, Chengdu 610065, China.

*Correspondence to:

Kun Yang

cookyoung@scu.edu.cn

Yonghong Li

liyonghong@scu.edu.cn

Supplementary Information Table of Contents

Supplementary documents

Supplementary document: Theory 4-7

Nomenclature 8-10

Supplementary tables

Supplementary Table S1. Primer sets of RT-LAMP for detecting SARS-CoV-2 genes. 11-12

Supplementary Table S2. Typical LAMP protocol. 13

Supplementary Table S3. Model parameters and relative abundance ($\boldsymbol{R_a}$) of the target gene (N) calculated based on the experimental data of mimic relative quantification via LAMP. 14

Supplementary Table S4. Model parameters and initial copy number of target nucleic acid determined via fitting the qPCR model (supplementary Eq. (S34)) with amplification curves. 15

Supplementary Table S5. Typical isothermal exponential DNA amplification technologies. 16

Supplementary figures

Supplementary Fig. S1. Quantitative doubling principle of target nucleic acid during IEA. 17

Supplementary Fig. S2. Designed experimental workflow. 18

Supplementary Fig. S3. Locations of primer sequences for LAMP on the SARS-CoV-2 sequence fragments in the pseudovirus (A) and the illustration of LAMP primer set design (B). 19

Supplementary Fig. S4. Quantifying target genes through amplification curve differentiation (d$\boldsymbol{c_t}$/d$\boldsymbol{t}$). 20

Supplementary Fig. S5. Comparison of two methods for determining doubling time. 21

Supplementary Fig. S6. Mimic relative quantification of N gene is SVNA. 22

Supplementary Fig. S7. The impacts of initial conditions and model parameters on qPCR were simulated via model calculation. 23

Supplementary Fig. S8. Experimental (markers) and simulated (lines) qPCR amplification curves of CPQ_056. 24

Supplementary Fig. S9. Theoretically calculated values of ln$\boldsymbol{R_a}$ versus $\boldsymbol{t_{P,s}}$-$\boldsymbol{t_{P,t}}$ under different $\boldsymbol{T_s}$ and $\boldsymbol{K_T}$ values. 25

Other supplementary material: MATLAB scripts and example experimental amplification curve datasets 26

Supplementary references 27-28

24

Supplementary Documents: Theory

1. Expressions of T^{θ} for various IEA mechanisms

The ideal doubling time T^{θ} can be derived for different IEA mechanisms, demonstrating the universality of the core kinetic model.

For Single-enzyme systems (e.g. LAMP):

$$T^{\theta} = \frac{S_a}{S_e} \tag{S1}$$

For dual-enzyme systems with two-step cycles (e.g. SDA/NEAR, RPA):

$$T^{\theta} = \frac{S_a}{S_e} + T_N \quad \text{(SDA/NERA)} \tag{S2}$$

For RPA, $\boldsymbol{T^{\theta}}$ can be expressed as:

$$T^{\theta} = \frac{S_a}{S_e} + T_R \quad \text{(RPA)} \tag{S3}$$

where, T_N = nicking time/cycle, T_R = recombination time/cycle.

For dual-enzyme systems with a single rate-limiting step (e.g. HDA):

$$T^{\theta} = \frac{S_a}{\min(S_e,\ S_H)} \tag{S4}$$

where, S_H = helicase unwinding rate (bp/min).

For multi-enzyme systems (e.g. NASBA/ TMA):

$$T^{\theta} = \frac{S_a}{S_{TR}} + 2\frac{S_a}{S_{RT}} + \frac{S_a}{S_{RH}} \tag{S5}$$

where, S_{TR} = RNA transcription rate (bp/min), S_{RT} = reverse transcription rate (bp/min), S_{RH} = RNase H hydrolysis rate (nt/min).

Note: For multi-step cycles, one rate-limiting reaction (typically strand extension) often dominates the kinetics. In this case, T^{θ} can be approximated by S_a/S_e (Eq. S1). This simplified form retains broad applicability across IEA mechanisms without significantly altering subsequent model deductions presented in the main text.

These expressions demonstrate that while different IEA techniques employ diverse biochemical mechanisms, their ideal doubling times T^0 can all be conceptually framed in terms of the fundamental parameters of amplicon size (S_a) and a rate parameter (e.g., S_e, S_H, *etc.*), thereby unified under the broader physical interpretation of our core model.

2. IEA quantification via standard curves

Under conditions of excess reporter molecules and non-saturated detection, the governing equation for the exponential amplification phase ($c_p >> c_t$, $\boldsymbol{\eta} = 1$) of IEA is given by:

$$I_P = \phi S_a c_P = \phi S_a c_0 2^{\frac{t_P}{T}} \tag{S6}$$

where the initial copy number ($\boldsymbol{N_0}$) is substituted with initial concentration ($\boldsymbol{c_0}$). Key parameters are defined as follows:

I_P: threshold signal intensity for positive detection, quantitatively measured via optical, fluorescence, turbidity, or electrochemical detector. I_P must be selected within the exponential amplification phase.

ϕ: signal intensity of unit reporter molecule.

S_a: apparent amplicon size (bp), denoting either the average amplicon length or the repeating unit size (e.g., for LAMP).

t_P: amplification time to reach the positive signal threshold (“time to positive”) (min).

c_0: initial target gene concentration (μM).

c_P: amplicon concentration at t_P (μM).

Taking the natural logarithm of Eq. S6 yields:

$$\ln I_P = \ln(\phi \cdot S_a) + \ln c_0 + \frac{\ln 2}{T} \cdot t_P \tag{S7}$$

Rearranged to express t_P as a function of $\ln c_0$:

$$\boldsymbol{t_P = -A \cdot \ln c_0 + B} \qquad \textbf{(S8)}$$

where

$$A = \frac{T}{\ln 2} \qquad \text{(S9)}$$

$$B = A \cdot \ln(\frac{I_P}{\phi \cdot S_a}) \qquad \text{(S10)}$$

This derivation establishes an inverse proportionality between the logarithm of initial target concentration ($\ln c_0$) and the time to positive detection (t_P). Plotting $\ln c_0$ versus t_P yields a linear relationship, from which the apparent doubling time T is derived via the slope A (Eq. S9).

Alternatively, quantification can be achieved through amplification curve differentiation (dc_t/dt), consistent with the approach described in (Hsieh et al., 2012). Here, t_P corresponds to the time at which dc_t/dt is maximized (Supplementary Fig. S4).

3. Relative quantification

For relative quantification, setting an identical threshold signal intensity (I_P) for both target (subscript t) and reference genes (subscript s) yields:

$$I_P = \phi S_{a,t} c_{0,t} 2^{\frac{t_{P,t}}{T_t}} = \phi S_{a,s} c_{0,s} 2^{\frac{t_{P,s}}{T_s}} \qquad \text{(S11)}$$

where $t_{P,t}$ and $t_{P,s}$ denote the time to positive (t_P) for the target and reference genes, respectively. The relative abundance of the target gene (R_a) is then derived as:

$$\boldsymbol{R_a = \frac{c_{0,t}}{c_{0,s}} = \frac{S_{a,s}}{S_{a,t}} \cdot 2^{\frac{t_{P,s}}{T_s} - \frac{t_{P,t}}{T_t}} = \frac{S_{a,s}}{S_{a,t}} \cdot 2^{\frac{T_t \cdot t_{P,s} - T_s \cdot t_{P,t}}{T_s \cdot T_t}}} \qquad \textbf{(S12)}$$

Defining the normalization factors:

$$K_S = \frac{S_{a,s}}{S_{a,t}} \qquad \text{(S13)}$$

$$\boldsymbol{K_T = \frac{T_s}{T_t}} \qquad \textbf{(S14)}$$

Eq. S12 simplifies to:

$$R_a = K_S \cdot 2^{\frac{t_{P,s} - K_T t_{P,t}}{T_s}} \quad \text{(S15)}$$

Taking the natural logarithms of both sides:

$$\ln R_a = \ln K_s + \frac{\ln 2}{T_s}(t_{P,s} - K_T t_{P,t}) \quad \text{(S16)}$$

This establishes a linear proportionality between the amplification time difference ($t_{P,s}$-$t_{P,t}$) and the logarithm of the initial concentration ratio ($\ln Ra$).

4. Mathematical model for qPCR

For comparison, we also derived the qPCR amplification model by extending Eq. 12 in the main text, as represented by the following equation.

$$N_{n+1} = (1 + \eta_n)N_n \quad \text{(S17)}$$

where N_n and N_{n+1} denote the amplicon copy numbers at cycle n and $n+1$ (copies/μL), respectively. The apparent amplification efficiency η_n (i.e., the template's primer-binding rate at cycle n) is defined as:

$$\eta_n = \eta^\theta \frac{c_{p,n}}{c_{p,n} + 2K_A \cdot c_n^v} \quad \text{(S18)}$$

Here, η^θ represents the maximal apparent amplification efficiency under primer-saturated conditions. The primer concentration at cycle n ($c_{p,n}$) is calculated by:

$$c_{p,n} = c_{p,0} - (c_n - c_0) \quad \text{(S19)}$$

Where $c_{p,0}$ is the initial primer concentration, and c_n (μM) is the amplicon concentration at cycle n, given by:

$$c_n = \frac{N_n}{R} \times 10^{12} \quad \text{(S20)}$$

Similarly, three model parameters (η^θ, K_A and v) are determined by fitting Eq. S17 to experimental qPCR amplification curves. Variation in the initial target copy number N_0 horizontally shifts amplification curves (Supplementary Fig. S7A). K_A exerts minimal influence on curve morphology (Supplementary Fig. S7B). The kinetics order v modulates the transition rate from exponential to plateau phase (Supplementary Fig.

S7C). The maximal efficiency η^{θ} governs the exponential-phase slope (Supplementary Fig. S7D).

Nomenclature

A	absolute slope of quantitative standard curve (min)
B	intercept of quantitative standard curve (min)
c_b	concentration of primer-template complex (μM)
c_n	the amplicon concentration at cycle n (μM)
c_P	amplicon concentration at t_P (μM)
c_p	primer concentration (μM)
c_t	total target concentration at time t (μM)
$c_{t,ss}$	single-stranded target concentration (μM)
$c_{t,ds}$	double-stranded target concentration (μM)
c_0	initial target gene concentration in reaction (μM)
$c_{0,i}$	initial target concentration in stock solution (μM)
I_P	threshold signal intensity for positive detection (arbitrary units)
K_A	Equilibrium constant for template self-annealing vs. primer-template binding (dimensionless)
K_S	amplicon size ratio: reference gene / target gene (dimensionless)
K_T	doubling time ratio: reference gene / target gene (dimensionless)
K_1	template self-annealing constant (μM^{-1})
K_2	primer-template binding constant (μM^{-1})
m	number of repeat units in structured amplicons
N_n	amplicon copy number at cycle n (copies/μL)
N_t	amplicon copy number at time t (copies/)

$N_{t+\Delta t}$	amplicon copy number at time t+Δt (copy/μL)
N_0	initial target copy number at t = 0 (copies/μL)
R	Avogadro's number (6.02 X 10^{23} mol^{-1})
R_a	relative abundance: target gene / reference gene (dimensionless)
S_e	DNA strand extension rate of (bp/min)
S_e^*	apparent strand extension rate: $S_e^* = \xi \cdot S_e$ (bp/min)
S_H	helicase unwinding rate of HDA (bp/min)
S_a	apparent amplicon size (bp)
S_p	average primer length (nt)
S_{RH}	RNase H hydrolysis rate (nt/min)
S_{RT}	reverse transcription rate (bp/min)
S_{TR}	RNA transcription rate (bp/min)
T	apparent doubling time: $T = \frac{S_a}{\xi \cdot S_e}$ (min)
T^θ	ideal doubling time: $T^\theta = \frac{S_a}{S_e}$ (min)
T_N	nicking time/cycle of SDA/NERA (min)
T_R	recombination time/cycle of RPA (min)
t	amplification time (min)
t_P	time to positive detection (min)
Δt	time increment (min)

Greek letters

η	template's primer-binding rate ($0 \leqslant \eta \leqslant 1$)
ξ	dimensionless primer-template binding efficiency: $\xi = \log_2(1+\eta)$

ϕ	signal intensity per reporter molecule (a.u./molecule)
λ	Poisson distribution parameter: $\lambda = \frac{\Delta t \cdot \ln 2}{T^{\theta}}$
v	amplicon concentration impact exponent (dimensionless)

Subscripts

a	amplification product
i	initial state (stock solution)
n	amplification cycle number
P	positive detection
p	primer
S	size-related parameter
s	reference gene
T	doubling time-related parameter
t	target gene or time t
t_0	any initial time point t_0
0	initial state (reaction mixture)

Supplementary Table S1. Primer sets of RT-LAMP and qPCR for detecting SARS-CoV-2 and crAssphage genes.

Target	Set name (Reference)	Primer	Sequence
S	S_1-2-2	F3	5’-CTGACAAAGTTTTCAGATCCTCAG-3’
	(Park et al., 2020)	B3	5’-AGTACCAAAAATCCAGCCTCTT-3’
		FIP	5’-TCCCAGAGACATGTATAGCATGGAATCAACTCAGGACTTGTTCTTACC-3
		BIP	5’-TGGTACTAAGAGGTTTGATAACCCTGTTAGACTTCTCAGTGGAAGCA-3’
		LF	5’-CCAAGTAACATTGGAAAAGAAA-3’
		LB	5’-GTCCTACCATTTAATGATGGTGTTT-3’
N	N_21	F3	5’-GCCAAAAGGCTTCTACGCA-3’
	(Park et al., 2020)	**B3[a]**	**5’-TTGCTCTCAAGCTGGTTCA~~A~~-3’**
		FIP	5’-TCCCCTACTGCTGCCTGGAGGCAGTCAAGCCTCTTCTCG-3’
		BIP	5’-TCTCCTGCTAGAATGGCTGGCATCTGTCAAGCAGCAGCAAAG-3’
		LF	5’-TGTTGCGACTACGTGATGAGGA-3’
		LB	5’-ATGGCGGTGATGCTGCTCT-3’
N1	Gene N-A	F3	5’-TGGCTACTACCGAAGAGCT-3’
	(Zhang et al., 2020)	B3	5’-TGCAGCATTGTTAGCAGGAT-3’
		FIP	5’-TCTGGCCCAGTTCCTAGGTAGTCCAGACGAATTCGTGGTGG-3’

		BIP	5’-AGACGGCATCATATGGGTTGCACGGGTGCCAATGTGATCT-3’
		LF	5’-GGACTGAGATCTTTCATTTTACCGT-3’
		LB	5’-ACTGAGGGAGCCTTGAATACA-3’
crAssphage	crAss_pp91[b]	F3	5’-ACTAAATCTTCCAAAACCAGG-3’
portal protein	(This work)	B3	5’-GTTACAACAAGAATTGTTGATGA-3’
gene		FIP	5’-GGTTCTCGTGCTACAAGCATATATCTACCATTATAAGGAAGTTTACCA-3’
		BIP	5’- TTCTAACGCTCTCATAAACTTGTGGACTTATCAGCTTAACCCTGC-3’
		LF	5’- CTCGTCCTATTGCTTACAATAGGAA-3’
		LB	5’- ACGCCACACCCATTCAATACTAAT-3’
crAssphage	CPQ_056	056F1	5’-CAGAAGTACAAACTCCTAAAAAACGTAGAG-3’
qPCR	(Stachler et al., 2017)	056R1	5’-GATGACCAATAAACAAGCCATTAGC-3’

[a] Compared with the original literature, the B3 primer used in this study was deleted the last adenosine.

[b] The LAMP primer set targeting crAssphage portal protein gene was design with PrimerExplorer V5 (http://primerexplorer.jp/lampv5e/index.html) based on the reference sequence of NCBI entry OP075736.1> UWI21366.1 (“Phage P22-like portal protein”).

Supplementary Table S2. Typical LAMP protocol. Incubate the following LAMP reaction mixture at 65°C (variable temperatures) for 30-60 min and detect the fluorescence intensity every 20 or 10 seconds

**Components**	**25 μL RXN**	**Final concentration**
10 x Isothermal amplification buffer	2.5 μL	1 x (contains 2 mM $MgSO_4$)
$MgSO_4$ (100 mM)	1.5 μL	6 mM (8 mM total)
dNTP Mix (10 mM)	3.5 μL	1.4 mM each
FIP/BIP primers (25 x)	1 μL	1.6 μM
F3/B3 primers (25 x)	1 μL	0.2 μM
LF/LB primers (25 x)	1 μL	0.4 μM
Bst 2.0 WarmStart DNA polymerase (8,000 U/mL)	1 μL	320 U/mL
BSA solution (20 mg/mL)	0.5 μL	0.4 mg/mL
LAMP dye (50 x)	0.5 μL	1 x
DNA sample	2 μL	>10 copies or more
Nuclease-free Water	to 25 μL	
Total reaction volume	25 μL	

Supplementary Table S3. Model parameters and relative abundance (***R_a***) of the target gene (N) calculated based on the experimental data of mimic relative quantification via LAMP (refer to Supplementary Fig. S6).

**Standard curves**			Conc. N[b]	**SVNA**		**dSVNA**		
Parameters	N	crAss_pp91	($c_0/c_{0,i}$)	T_N (min)	R_a (N/crAss_pp91)	T_N (min)	R_a (N/crAss_pp91)	**Dev_R_a%**
A	0.341	0.563	10^0		5.631E+00		5.677E+00	0.82
T	0.237	0.390	10^{-1}		9.472E-01		7.284E-01	-23.10
(min)			10^{-2}	0.286	9.496E-02	0.258	6.689E-02	-29.56
K_T[a]	**0.6065**[a]		10^{-3}		7.168E-03		4.869E-03	-32.07
(N/crAss_pp91)			10^{-4}		9.441E-04		7.526E-04	-20.28

[a] ***K_T*** value is considered stable at the same temperature and is little affected by polymerase activity and inhibitors. The ***T*** values of crAss_pp91 in SVNA and dSVNA were calculated with the ***K_T*** and corresponding ***T_N*** according to Eq. S27, respectively.

[b] The concentration of N gene fragment is expressed as their dilution ratio ($c_0/c_{0,i}$) before being spiked in SVNA.

Supplementary Table S4. Model parameters and initial copy number of target nucleic acid determined via fitting the qPCR model (supplementary Eq. (S34)) with amplification curves (Supplementary Fig. S8)

**Run**	**N_0 (copies/μL)**	$\boldsymbol{K_A}$	$\boldsymbol{v}$	$\boldsymbol{\eta^{\theta}}$	***MSE* X 10^4**
Single-1[a]	9.69E+07	11.460	2.183	0.777	0.089
Single-2[a]	7.83E+06	5.943	1.870	0.836	0.285
Single-3[a]	6.99E+05	8.660	1.853	0.845	0.045
Single-4[a]	1.12E+05	9.087	2.083	0.795	0.059
Single-5[a]	1.25E+04	11.127	2.127	0.793	0.070
Single-6[a]	1.41E+03	10.245	2.069	0.808	0.048
All[b]	7.19E+07	7.986	1.939	0.841	1.534

a: Model parameters ($\boldsymbol{K_A}$, $\boldsymbol{v}$ and $\boldsymbol{\eta^{\theta}}$) and initial copy number of target nucleic acid ($\boldsymbol{N_0}$) obtained by fitting a single amplification curve one by one.

b: Model parameters and initial copy number of target nucleic acid (in the amplification of the highest initial concentration) obtained by fitting all 6 amplification curves as a whole.

Supplementary Table S5. Typical isothermal exponential DNA amplification technologies.

Name	Reaction temperature (ºC)	Amplicon size (bp or nt)	Reported doubling time & literatures of data source（*T*, min）	DNA product	Mechanism comments	Shortcoming	References
Loop-mediated isothermal amplification (**LAMP**)	**65**	<250	0.301 (Nixon et al., 2014)	Long, branched	A strand-displacing DNA polymerase (Bst DNA pol), specially designed primers form “loop” structures.	4-6 primers	(Nagamine et al., 2002; Notomi et al., 2000)
Whole genome amplification (**WGA**)	30	N/A	N/A	Long, branched	Amplify an entire genome, enable sequencing of single-cell genomic DNA.	Not suitable for nucleic acid detection; low amplification temperature cannot ensure the specialty of the amplification.	(Ahrabi et al., 2010)
Strand displacement amplification (**SDA**)<br>Nicking enzyme amplification reaction (**NEAR**)	37	<100	5.3 (Walker et al., 1992b)	Short, discrete	A strand-displacing DNA polymerase, a strand-limited restriction endonuclease or nicking enzyme.	Special restriction endonuclease or nicking enzyme needed.	(Walker et al., 1992a; Walker et al., 1992b)
Helicase-dependent amplification (**HDA**)	**65**	<150	3.0 (Vincent et al., 2004)<br>1.17 (Barreda-Garcia et al., 2015)	Short, discrete	Employs the double-stranded DNA unwinding activity of a helicase to separate strands.	N/A	(Vincent et al., 2004)
Recombinase polymerase amplification (**RPA**)<br>Strand-invasion based amplification (**SIBA**)	37	<1,000	1.41 (Piepenburg et al., 2006)	Short, discrete	A recombinase enzyme helps primers invade into double-stranded DNA.	Low amplification temperature	(Hoser et al., 2014; Piepenburg et al., 2006)
Nucleic acid sequences-based amplification (**NASBA**)<br>Transcription mediated amplification (**TMA**)	40-55	<150	1.09 (Ju et al., 2021)	Short, discrete	One primer includes the promoter sequence for T7 RNA polymerase; a reverse transcriptase and RNase H are included in the reaction.	Low amplification temperature	(Compton, 1991)
Exponential amplification reaction (**EXPAR**)	**50-60**	**15-20**	0.687 (Carter et al., 2021)	Short, discrete	A strand-displacing DNA polymerase (Bst DNA pol), a strand-limited restriction endonuclease or nicking enzyme, a sequence-specific triggering mechanism.	Non-specific amplification	(Carter et al., 2021; Van Ness et al., 2003)

N/A: Not available.

Supplementary Fig. S1. Quantitative doubling principle of target nucleic acid during IEA. (A) Ideally, after each doubling time ($T^{\theta} = \frac{S_a}{S_e}$), the copy number of the target gene fragment will double. While, under non-ideal conditions with primer-template binding efficiency $\eta < 100\%$, after any period of time ($t = n\frac{S_a}{S_e}$), the copy number of the amplification product (N_t) is $(1+\eta)^n N_0$. (B) The schematic diagram of the principle of IEA omits the description of the mechanism of double strand unwinding. There are differences in the strand unwinding mechanism among different IEAs, but the quantitative mechanism of the exponential amplification is similar. (C) The strands extension catalyzed by the nucleic acid polymerase promotes the primer-template annealing equilibrium and brings the reaction into the next amplification cycle.

A

Time (t)	The copy number of target gene under ideal conditions (η=100%)	The copy number under non-ideal conditions with primer-template binding efficiency of η<100%
$t = 0$	N_0	N_0
$t = \frac{S_a}{S_e}$	$2N_0$	$(1+\eta)N_0$
$t = 2\frac{S_a}{S_e}$	2^2N_0	$(1+\eta)^2N_0$
…	…	…
$t = n\frac{S_a}{S_e}$	2^nN_0	$(1+\eta)^nN_0$
**Doubling time**	$T^{\theta} = \frac{S_a}{S_e}$	$T = \frac{T^{\theta}}{\xi}$ ($\xi = \log_2(1+\eta)$)

B

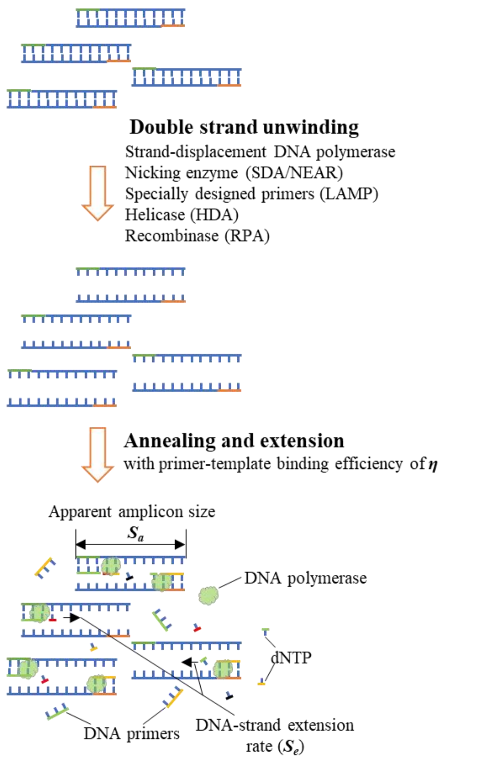

C

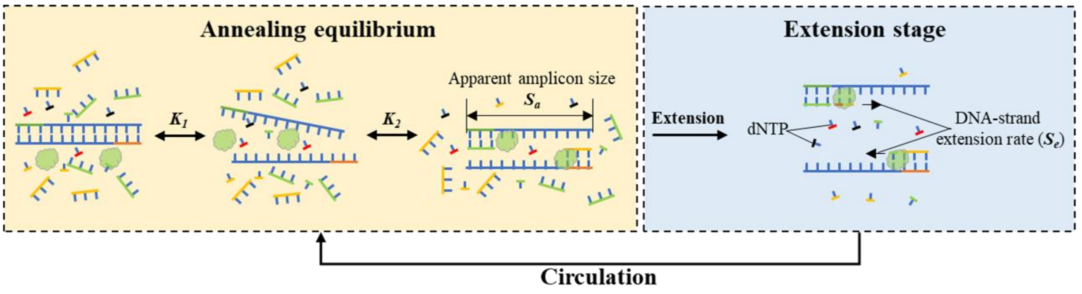

Supplementary Fig. S2. Designed experimental workflow. The PCR amplification products with LAMP outer primer pair (F3 and B3) were serially diluted as the templates of LAMP to construct the quantification standard curves. The effects of enzyme concentration, temperature and inhibitor on the LAMP amplification efficiency were evaluated to verify the rationality of the quantification mathematical model.

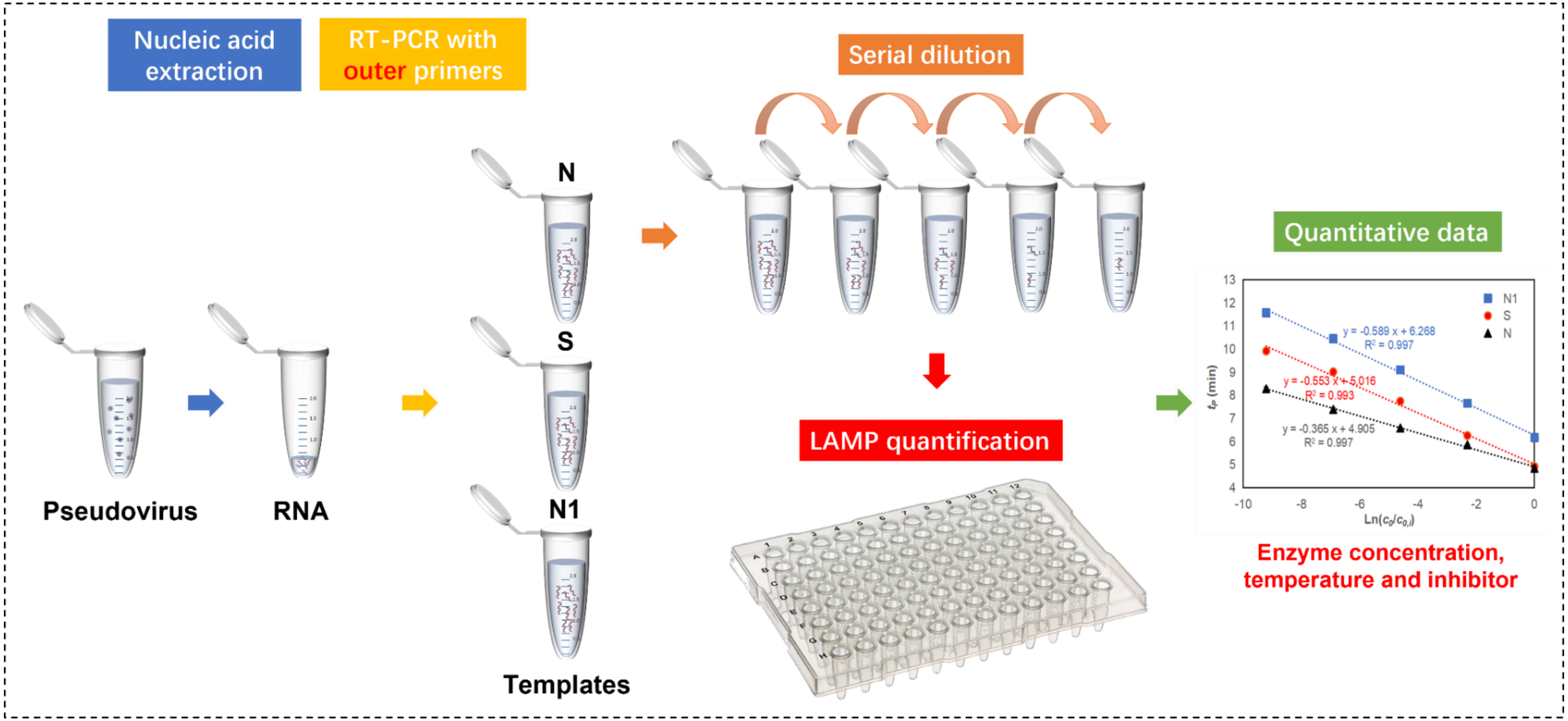

Supplementary Fig. S3. Locations of primer sequences for LAMP on the SARS-CoV-2 sequence fragments in the pseudovirus (A) and the illustration of LAMP primer set design (B).

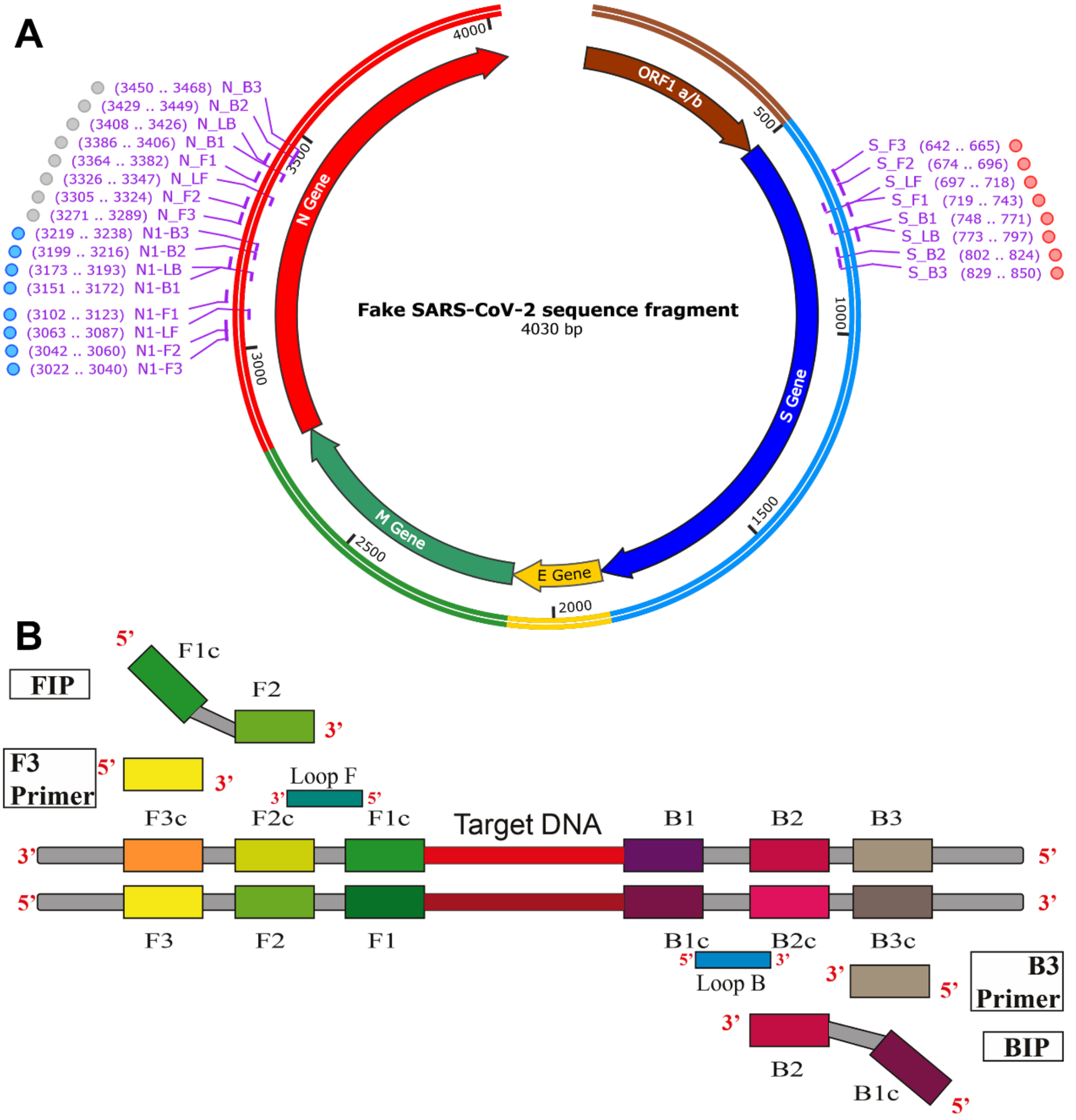

Supplementary Fig. S4. Quantifying target genes through amplification curve differentiation (d$\boldsymbol{c_t}$/d$\boldsymbol{t}$), which is similar with the documented method (Hsieh et al., 2012). The embedded diagram therein is the standard curve for quantification, plotting N_0 versus t_P. The time when dc_t/dt reaches its maximum value is defined as t_P. The slop of the line is equal to $\frac{T}{\ln 2}$, and the value of T is calculated to be 0.246 min.

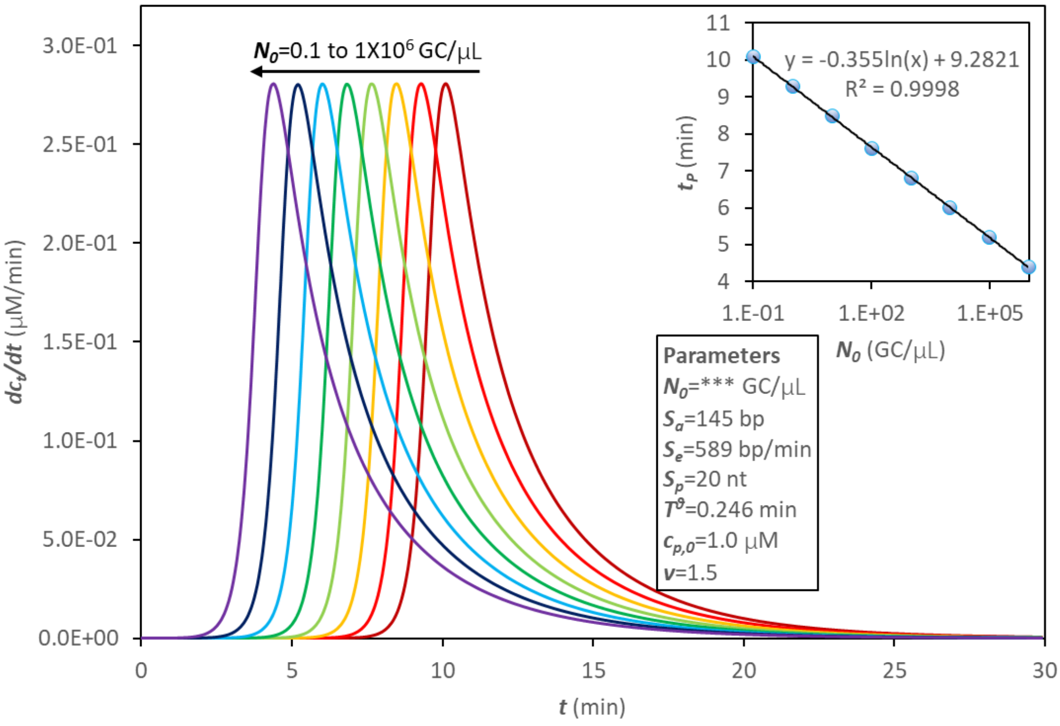

Supplementary Fig. S5. Comparison of two methods for determining doubling time.

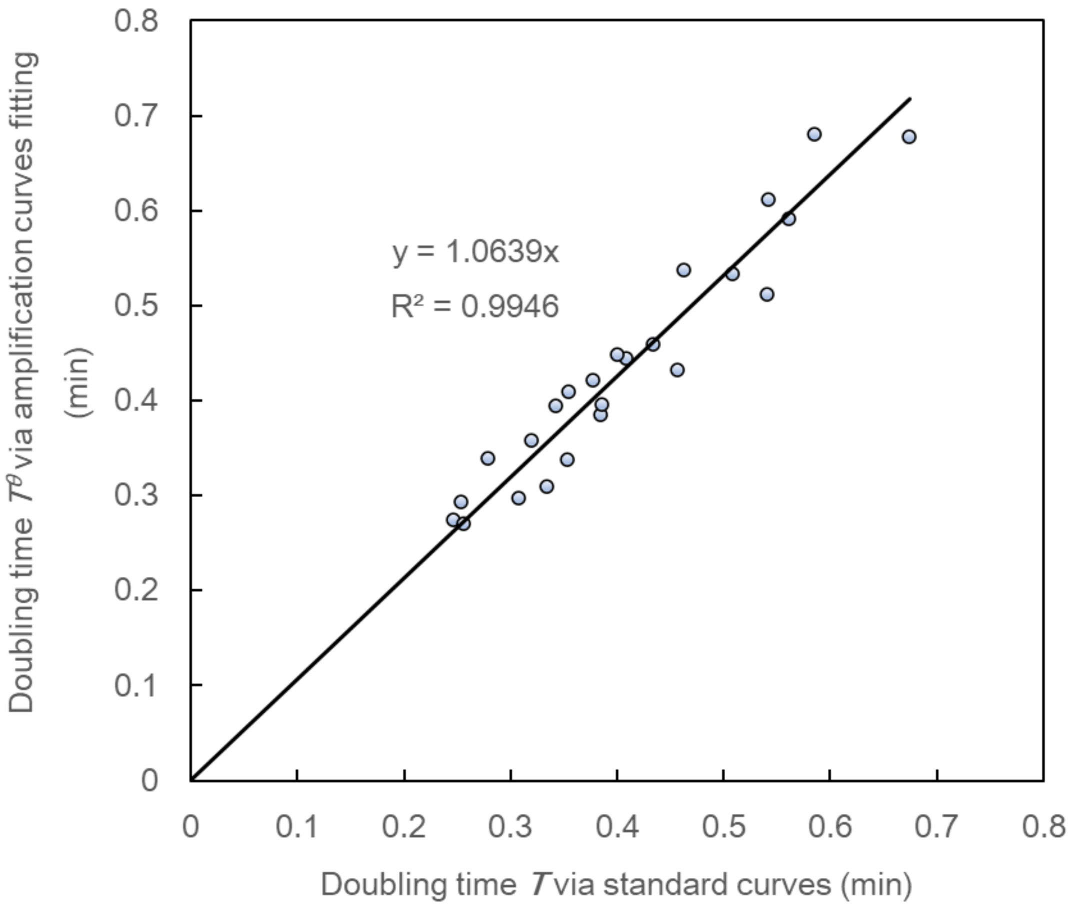

Supplementary Fig. S6. Mimic relative quantification of N gene is SVNA. The outer-primer PCR/RT-PCR amplicon of crAss_pp91/N was serially diluted to construct the LAMP quantification standard curves (A). Serially diluted SARS-CoV-2 N gene fragment was serially diluted and spiked in SVNA to mimic the relative quantification of viral nucleic acid in sewage via LAMP (B). To verify the robustness of relative quantification, the SVNA samples were further diluted and quantified with LAMP again (C).

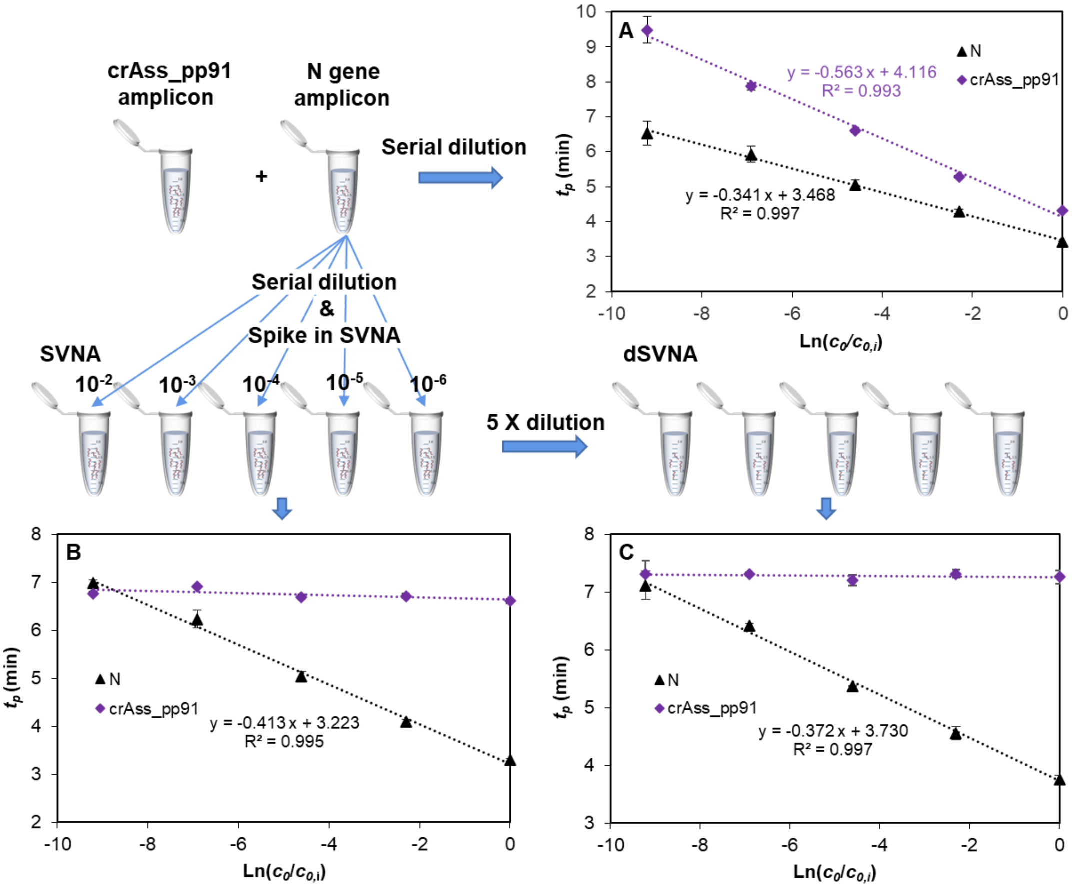

Supplementary Fig. S7. The impacts of initial conditions and model parameters on qPCR were simulated via model calculation.

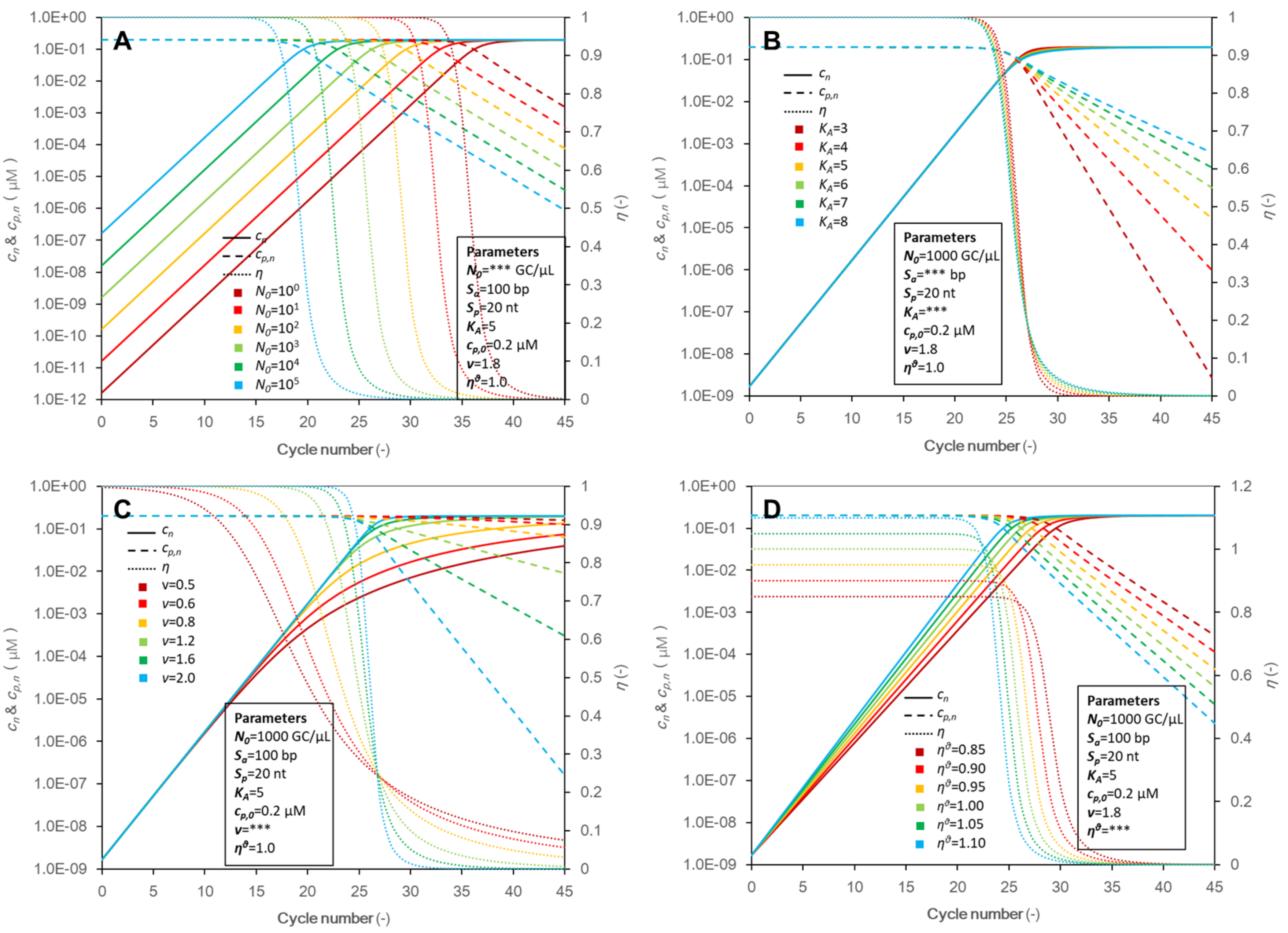

Supplementary Fig. S8. Experimental (markers) and simulated (lines) qPCR amplification curves of CPQ_056. $\boldsymbol{N_0}$ =6.27X10^7-6.27X10^2 (copies/ μL), $\boldsymbol{K_A}$=8.690, $\boldsymbol{v}$=1.965 and $\boldsymbol{\eta^\theta}$=0.846. The results of fitting a single amplification curve one by one (dotted lines) or fitting all 6 amplification curves as a whole (solid lines) are shown in both linear (A) and semi-logarithmic (B) coordinates.

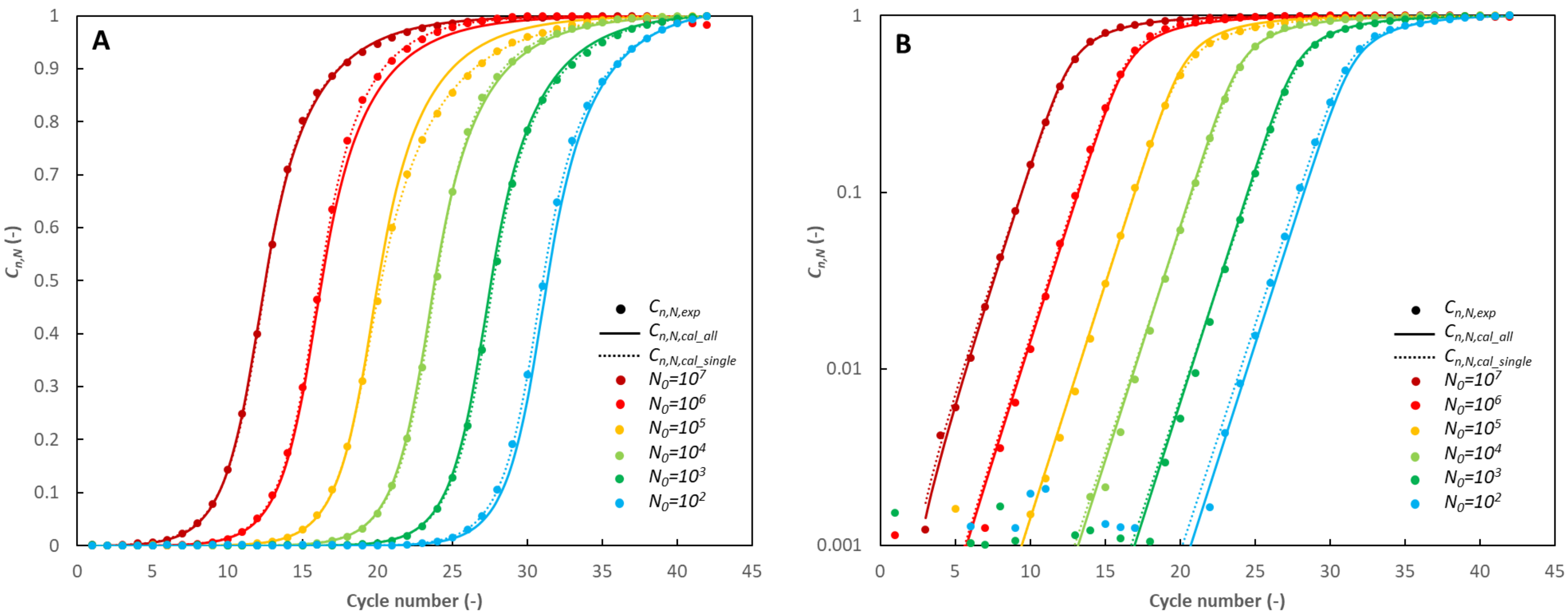

Supplementary Fig. S9. Theoretically calculated values of ln$\boldsymbol{R_a}$ versus $\boldsymbol{t_{P,s}}$-$\boldsymbol{t_{P,t}}$ under different $\boldsymbol{T_s}$ and $\boldsymbol{K_T}$ values. The results are displayed as 3D surfaces (A) and 2D lines (B). The value of ln$\boldsymbol{R_a}$ is proportional to the △$\boldsymbol{t_P}$.

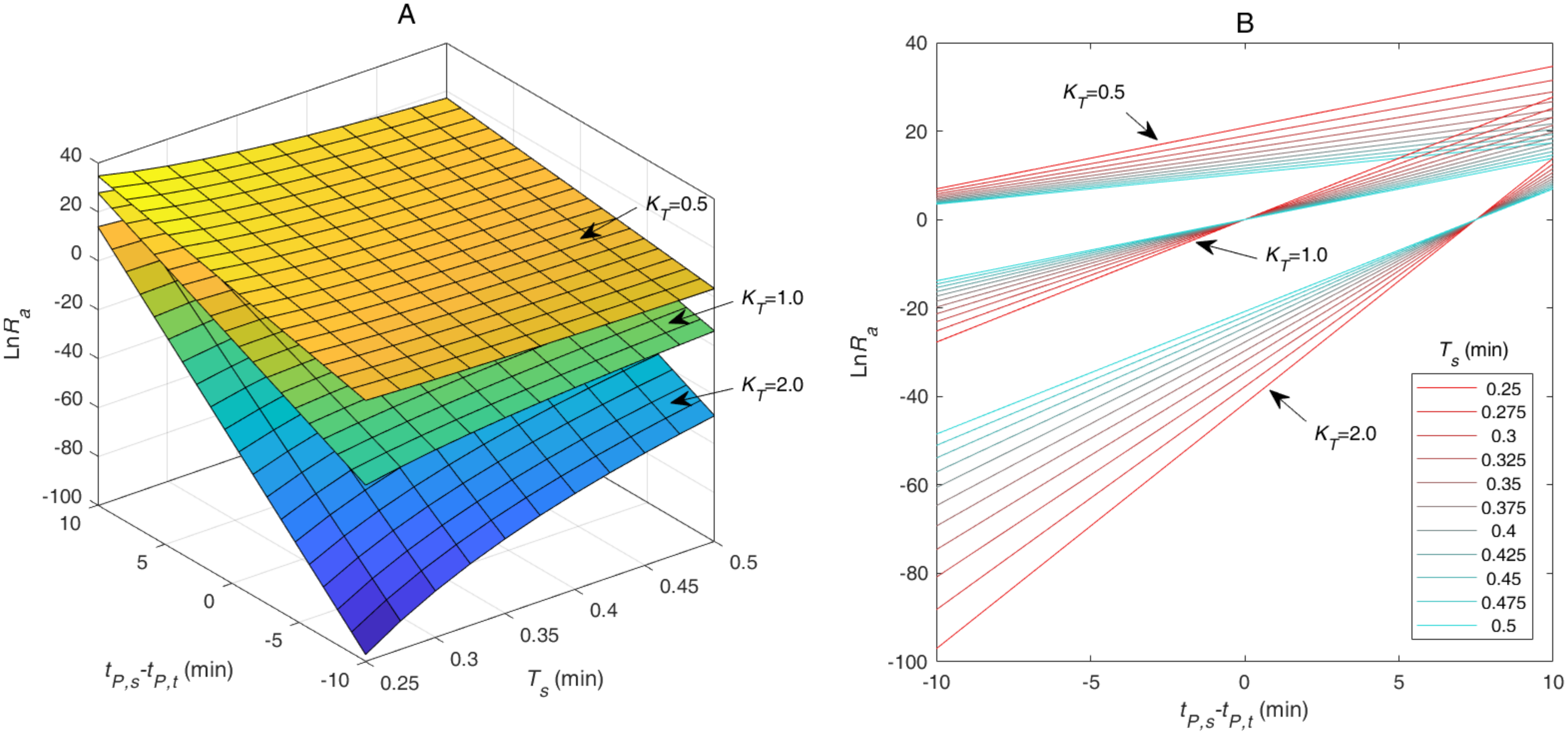

Other supplementary material: MATLAB scripts and example experimental amplification curve datasets

Supplementary MATLAB implementation scripts and example experimental amplification curve datasets are bundled in the compressed file **MATLAB scripts & Exp. data.rar** to enable reproducibility testing:

The IEA model can be fitted to raw LAMP amplification curve data (**67-1-S.csv**) by executing the MATLAB script **IEAParameterfitN.m**. Optimized model parameters were returned in vector **Para**.

The qPCR model can be fitted to raw crAssphage qPCR amplification curve data (**crAssphage.csv**) by executing the MATLAB script **qPCRParameterfitN.m**. Optimized model parameters were returned in vector **Para**.

Supplementary references

Ahrabi, S.M., Farajnia, S., Rahimi-Mianji, G., Saheb, S.M. and Nejati-Javaremi, A. 2010. Whole genome amplification: Use of advanced isothermal method. Afr J Biotechnol 9(54), 9248-9254.

Barreda-Garcia, S., Gonzalez-Alvarez, M.J., de-Los-Santos-Alvarez, N., Palacios-Gutierrez, J.J., Miranda-Ordieres, A.J. and Lobo-Castanon, M.J. 2015. Attomolar quantitation of Mycobacterium tuberculosis by asymmetric helicase-dependent isothermal DNA-amplification and electrochemical detection. Biosens Bioelectron 68, 122-128.

Carter, J.G., Orueta Iturbe, L., Duprey, J.H.A., Carter, I.R., Southern, C.D., Rana, M., Whalley, C.M., Bosworth, A., Beggs, A.D., Hicks, M.R., Tucker, J.H.R. and Dafforn, T.R. 2021. Ultrarapid detection of SARS-CoV-2 RNA using a reverse transcription-free exponential amplification reaction, RTF-EXPAR. Proc Natl Acad Sci U S A 118(35).

Compton, J. 1991. Nucleic acid sequence-based amplification. Nature 350(6313), 91-92.

Hoser, M.J., Mansukoski, H.K., Morrical, S.W. and Eboigbodin, K.E. 2014. Strand Invasion Based Amplification (SIBA(R)): a novel isothermal DNA amplification technology demonstrating high specificity and sensitivity for a single molecule of target analyte. PLoS One 9(11), e112656.

Hsieh, K., Patterson, A.S., Ferguson, B.S., Plaxco, K.W. and Soh, H.T. 2012. Rapid, sensitive, and quantitative detection of pathogenic DNA at the point of care through microfluidic electrochemical quantitative loop-mediated isothermal amplification. Angew Chem Int Ed Engl 51(20), 4896-4900.

Ju, Y., Kim, H.Y., Ahn, J.K. and Park, H.G. 2021. Ultrasensitive version of nucleic acid sequence-based amplification (NASBA) utilizing a nicking and extension chain reaction system. Nanoscale 13(24), 10785-10791.

Nagamine, K., Hase, T. and Notomi, T. 2002. Accelerated reaction by loop-mediated isothermal amplification using loop primers. Mol Cell Probes 16(3), 223-229.

Nixon, G.J., Svenstrup, H.F., Donald, C.E., Carder, C., Stephenson, J.M., Morris-Jones, S., Huggett, J.F. and Foy, C.A. 2014. A novel approach for evaluating the performance of real time quantitative loop-mediated isothermal amplification-based methods. Biomol Detect Quantif 2, 4-10.

Notomi, T., Okayama, H., Masubuchi, H., Yonekawa, T., Watanabe, K., Amino, N. and Hase, T. 2000. Loop-mediated isothermal amplification of DNA. Nucleic Acids Res 28(12), E63.

Park, G.S., Ku, K., Baek, S.H., Kim, S.J., Kim, S.I., Kim, B.T. and Maeng, J.S. 2020. Development of Reverse Transcription Loop-Mediated Isothermal Amplification Assays Targeting Severe Acute Respiratory Syndrome Coronavirus 2 (SARS-CoV-2). J Mol Diagn 22(6), 729-735.

Piepenburg, O., Williams, C.H., Stemple, D.L. and Armes, N.A. 2006. DNA detection using recombination proteins. PLoS Biol 4(7), e204.

Stachler, E., Kelty, C., Sivaganesan, M., Li, X., Bibby, K. and Shanks, O.C. 2017. Quantitative CrAssphage PCR Assays for Human Fecal Pollution Measurement. Environ Sci Technol 51(16), 9146-9154.

Van Ness, J., Van Ness, L.K. and Galas, D.J. 2003. Isothermal reactions for the amplification of oligonucleotides. Proc Natl Acad Sci U S A 100(8), 4504-4509.

Vincent, M., Xu, Y. and Kong, H. 2004. Helicase-dependent isothermal DNA amplification.

EMBO Rep 5(8), 795-800.

Walker, G.T., Fraiser, M.S., Schram, J.L., Little, M.C., Nadeau, J.G. and Malinowski, D.P. 1992a. Strand displacement amplification--an isothermal, in vitro DNA amplification technique. Nucleic Acids Res 20(7), 1691-1696.

Walker, G.T., Little, M.C., Nadeau, J.G. and Shank, D.D. 1992b. Isothermal in vitro amplification of DNA by a restriction enzyme/DNA polymerase system. Proc Natl Acad Sci U S A 89(1), 392-396.

Zhang, Y., Ren, G., Buss, J., Barry, A.J., Patton, G.C. and Tanner, N.A. 2020. Enhancing colorimetric loop-mediated isothermal amplification speed and sensitivity with guanidine chloride. Biotechniques 69(3), 178-185.
